# Cannabinoids, Inner Ear, Hearing, and Tinnitus: A Neuroimmunological Perspective

**DOI:** 10.3389/fneur.2020.505995

**Published:** 2020-11-23

**Authors:** Paola Perin, Alex Mabou Tagne, Paolo Enrico, Franca Marino, Marco Cosentino, Roberto Pizzala, Cinzia Boselli

**Affiliations:** ^1^Department of Brain and Behavioural Sciences, University of Pavia, Pavia, Italy; ^2^University of Insubria, Varese, Italy; ^3^University of Sassari, Sassari, Italy; ^4^Department of Molecular Medicine, University of Pavia, Pavia, Italy; ^5^Department of Drug Sciences, University of Pavia, Pavia, Italy

**Keywords:** cannabinoids, tinnitus, auditory, neuroimmune, hearing

## Abstract

Cannabis has been used for centuries for recreational and therapeutic purposes. Whereas, the recreative uses are based on the psychotropic effect of some of its compounds, its therapeutic effects range over a wide spectrum of actions, most of which target the brain or the immune system. Several studies have found cannabinoid receptors in the auditory system, both at peripheral and central levels, thus raising the interest in cannabinoid signaling in hearing, and especially in tinnitus, which is affected also by anxiety, memory, and attention circuits where cannabinoid effects are well described. Available studies on animal models of tinnitus suggest that cannabinoids are not likely to be helpful in tinnitus treatment and could even be harmful. However, the pharmacology of cannabinoids is very complex, and most studies focused on neural CB1R-based responses. Cannabinoid effects on the immune system (where CB2Rs predominate) are increasingly recognized as essential in understanding nervous system pathological responses, and data on immune cannabinoid targets have emerged in the auditory system as well. In addition, nonclassical cannabinoid targets (such as TRP channels) appear to play an important role in the auditory system as well. This review will focus on neuroimmunological mechanisms for cannabinoid effects and their possible use as protective and therapeutic agents in the ear and auditory system, especially in tinnitus.

## Introduction

**Figure 1:**
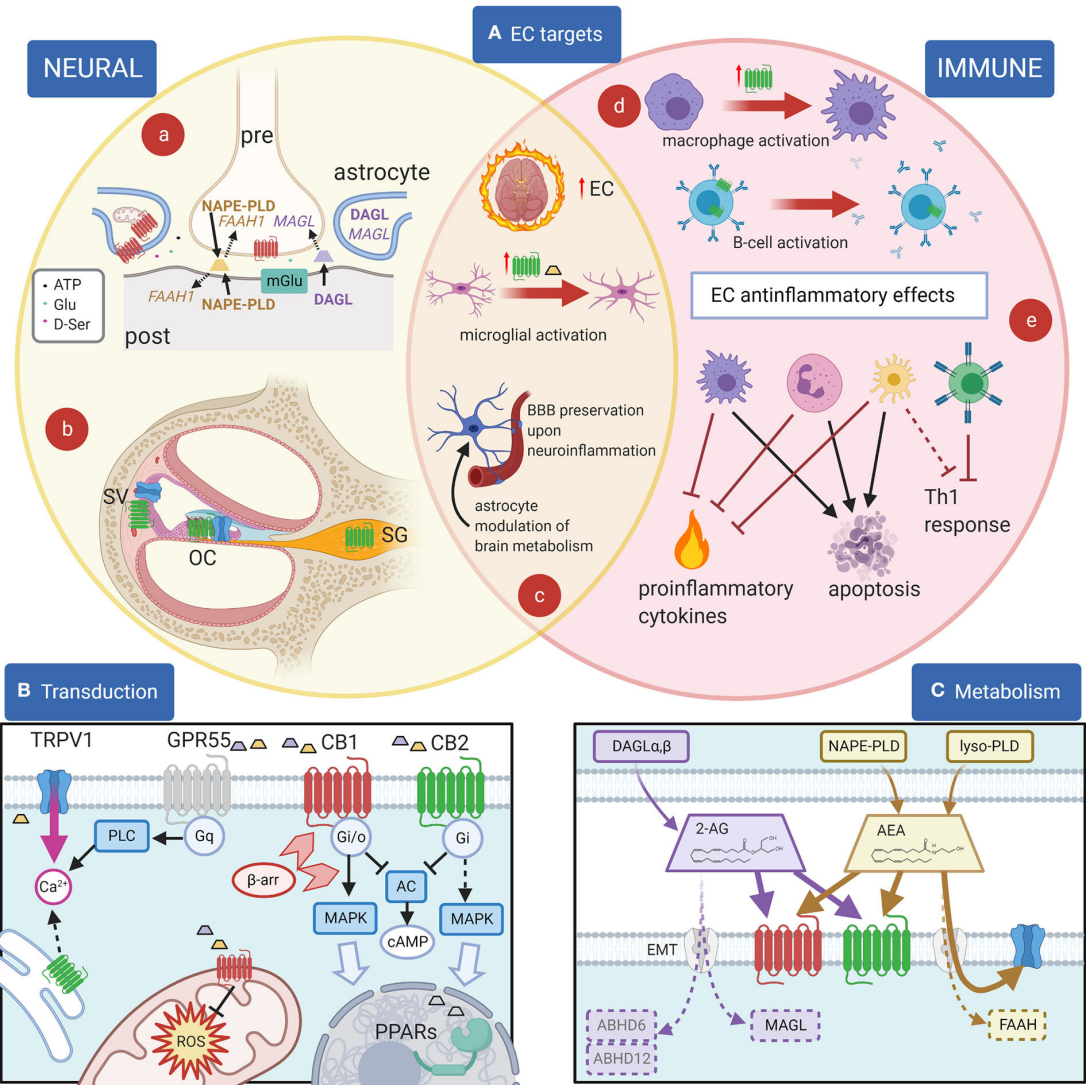
EC and their effects. **(A)**: Principal EC targets in neural and immune systems, and in the cochlea. (a) In most brain areas, 2-AG (purple) is synthetized by DAGL-α in neuronal dendrites and somata and catabolized by MAGL in presynaptic terminals, where CB1R (red) are also present. 2-AG is produced postsynaptically in a Ca^2+^-dependent way upon activation of metabotropic receptors (blue) and inactivated presynaptically near its target (1). For AEA (yellow), on the other hand, the biosynthetic enzyme NAPE-PLD is both pre- and postsynaptic, and the catabolic enzyme FAAH-1 is predominantly postsynaptic (1). Astrocytes are also involved in synaptic effects through an EC modulation of gliotransmission, and in addition EC effects on astrocyte mitochondria contribute to neuronal metabolism regulation. (b) In the cochlea, CB2R (green) are found in the organ of Corti (OC), basal cells of the stria vascularis (SV) and spiral ganglion (SG), whereas TRPV1 channels (blue) are found in the organ of Corti and marginal cells of the stria vascularis. (c) During neuroinflammation, several changes are seen in the EC system. The overall EC production increases. Activated microglia increases AEA production (yellow trapezoid) and CB2R expression (green). Astrocytes become activated and BBB is affected (both effects are counteracted by EC responses). (d) Cell activation may change CB2R expression as in macrophages (purple) (2) or CB2R subcellular localization as in B lymphocytes (blue) (3). (e) Anti-inflammatory EC responses in immune cells include the block of Th1 responses due to direct effects on T cells (green) and indirect effects on dendritic cells (yellow), apoptosis induction on several cell types, and the inhibition of proinflammatory cytokines and factors. **(B)**: Principal EC receptors and their main intracellular pathways. Both 2-AG (purple trapezoid) and AEA (yellow trapezoid) act on CB1R and CB2R, which are class A GPCRs (4) coupled to Gi/o G-proteins (5, 6), reducing cAMP concentration (7). β-Arrestin binding (light red arrowheads) induces CBR internalization and switches receptor coupling, especially for CB1, also activating MAP kinase pathways (8–10) linked to nuclear effects (large light blue arrow). MAPK pathways are also activated through Gi βγ action (dotted line), both by CB1R and by CB2R (11). Functional CBRs have been also found in intracellular compartments such as the outer mitochondrial membrane (12), where they modulate cell energetic balance (13), and ROS production (14) or endoplasmic reticulum, where they may induce Gq-related Ca2+ release from intracellular stores (3). Moreover, ECs or related lipids activate nuclear PPARs (15). TRPV1 channels are often colocalized with CB1R and/or CB2R and activated by cytoplasmic ECs (16), increasing cytosolic Ca2+. Finally, orphan receptors may activate other pathways, e.g., GPR55 is linked to Gq-PLC and therefore contributes to cytosolic Ca2+ increase. Most cells only express a subset of these pathways. **(C)**: Metabolism of 2-AG and anandamide (AEA). 2-AG (purple) is produced from DAG by DAG lipases (DAGLα and β). Biosynthesis of AEA (yellow) is more complex and may involve hydrolysis of NAPE membrane phospholipids by NAPE-PLD (which directly generates AEA) or sequential action of several enzymes (not shown), followed by a lyso-PLD (17). Although lipophilic, ECs have membrane transport mechanisms (EMT, light gray) (18). EC binding sites on CB1 (red) and CB2 (green) are extracellular, whereas on TRP channels (blue) the site is intracellular. Degradation of AEA is mainly due to FAAH, whereas 2-AG is primarily degraded by MAGL, and less by ABHD6 and ABHD12 (19). Created with Biorender.

Endocannabinoids (ECs; Figure 1) are a class of ubiquitous endogenous lipids regulating essential processes ranging from energy balance, to pain, to motor control, and involved in pathologies as diverse as (among others) schizophrenia, glaucoma, multiple sclerosis, and obesity (20). In the CNS, ECs influence synaptic plasticity (21, 22), modulate neuroinflammation (23), and affect neurogenesis (24) and may also affect neuronal activity by binding to neurotransmitter receptors and ion channels (25). These cellular effects are reflected in the EC modulation of several brain functions, including fear and anxiety (26), or memory (27). Overall, the standard arrangement in the brain appears to be the presence of multiple EC pathways affecting the same circuits, often with different or even opposing effect.

In the immune system, ECs affect cell proliferation, migration, differentiation, cytokine production, and apoptosis (28). The two responses, immune and neural, interact in neuroinflammation, where ECs play major roles (29). Earlier studies suggested that neural effects of cannabinoids are mediated by CB1R activation (30) whereas immune effects are mediated by CB2R (31). However, it is important to stress that the separation of these biological actions is not as clear-cut as initially suggested (32), and other receptors can also be activated by ECs. The dizzying complexity of cannabinoid pharmacology (see Supplementary Tables 1, 2) requires a deep knowledge of the precise “fingerprint” of the molecular pathways affected by each compound, in each organ and each species, to dissect its effects.

### Cannabinoid Pharmacology

The pharmacology of cannabinoids is very complex, for several reasons. First, more than one hundred phytocannabinoids (33) and at least 13 ECs (25) have been identified. Second, their lipidic nature makes unraveling their molecular interactions more difficult than for conventional transmitters (34). Third, CBRs are connected to several intracellular pathways (Figure 1B) and may produce different (even opposite) results depending on the particular ligand and its concentration (8, 9, 35) and on the cell repertoire of signal transduction molecules (36).

ECs are one of the four families of bioactive lipids (together with classical eicosanoids, SPMs, and lysoglycerophospholipids/sphingolipids), which are generated from PUFA precursors esterified into membrane lipids (37). The EC system includes CBRs, their endogenous ligands, and the proteins involved in EC formation, transport, and degradation.

The first discovered and best-characterized ECs are AEA and 2-AG (38–40). Several other EC lipid mediators (41–43) [and a family of EC peptides, named “pepcans” (44)] have also been described (see also Supplementary Table 1), but their endogenous functions have been less characterized.

ECs (Figure 1C) are produced “on demand” from membrane lipids by several Ca^2+^-dependent enzymes (17), and metabolic pathways for production, transport, and degradation differ for the various ECs, making it possible for cells to tailor their local EC repertoire (45) by regulating their local concentrations through modulation of their biosynthesis, transport, and degradation (46). Once released, ECs are rapidly deactivated by intracellular enzymes (47): AEA by FAAH1 and 2 [the latter not expressed in rodents (1)], and 2-AG mainly by MAGL, and less by ABHD6 and ABHD12 (19). In addition, ECs may be transformed in non-EC bioactive metabolites [e.g., by COX-2 (48)].

ECs (Figure 1B) bind and activate two specific G-protein-coupled cannabinoid receptors, CB1R and CB2R (49–51), plus additional targets (52), such as TRP channels (53), PPARs (54–57), and “orphan” G-protein coupled receptors such as GPR18 and GPR55 (58, 59). Most EC are able to activate both CB1R and CB2R, although with different potency and effects (60), whereas nonclassical targets may interact with limited EC subsets (Supplementary Table 1) and also non-EC ligands. A clear example is TRPV1, which is activated by AEA binding to a cytoplasmic site (16) but is also sensitive to other stimuli such as heat, vanilloids, protons, N-acyl amides, and arachidonic acid derivatives (61).

CB1R and CB2R are class A (rhodopsin-like) GPCRs (4), and both couple to Gi/o G-proteins (5, 6), reducing cAMP concentration (7). However, the coupling between CBRs and biochemical pathways is complex and context-dependent. First, CB1Rs may form homo- or heterodimers with other GPCRs (62), such as (among others) CB2Rs (63), A2A adenosine receptors (64), D2 dopamine receptors (65), μ opioid receptors (66), and orexin-1 receptors (67), whereas CB2R may dimerize with the CXCR4 chemokine receptor (68) and GPR55 (69). The presence of CB1R/CB2R heteromers makes it impossible to clearly separate the biological responses of CB1R and CB2R *in vivo*.

Second, CBRs show dimerization- and agonist-biased response, due to conformation-dependent binding by β-arrestins (10). Besides blocking interactions with Gi/o proteins, β-arrestin effects include CBR internalization and ERK pathway/Gs protein activation (9), so that cAMP levels may increase instead of decreasing depending on CBR receptor bias. Receptor coupling flexibility appears more limited (although not absent) for CB2Rs, which mainly activate Gi proteins, whereas CB1Rs may couple to Go, Gs, Gq, and G12/13, thus activating a very diverse network of responses (8). Both receptors are in addition able to activate ER stress pathways linked to autophagy (70).

The β-arrestin-dependent internalization of plasma membrane CBRs is linked to receptor degradation (9); however, functional CBRs have been found in the outer mitochondrial membrane (12), and in the endoplasmic reticulum, endosomes, lysosomes, and nuclear membrane (3). Subcellular localization affect CB-related responses: mitochondrial localization allows CBRs to modulate cell energetic balance (13), and ROS production (14), whereas endolysosomal localization is correlated with inflammation and phagocytosis (71). Moreover, intracellular receptor sites will be inaccessible to membrane-impermeant cannabinoid agonists and antagonists.

Besides G-protein-coupled receptors, TRP nonselective cation channels are being increasingly recognized as an integral part of the EC system (ionotropic EC receptors): six of the 28 TRP channels are sensitive to cannabinoids (53). Among these, TRPV1 is the most studied, mainly due to its expression in nociceptors and role in pain-related processes: TRPV1 channels are colocalized with CB1R and/or CB2R in several types of cells, and TRPV1 block or desensitization underlies analgesia (72); the analgesic and antihyperalgesic effects of phytocannabinoids are, at least in part, mediated by this channel (53).

### EC System in the Brain

CBRs are expressed in most tissues of the body (73) and are by far the most abundant type of G-protein-coupled receptors in the mammalian brain (74). CB1R is predominantly expressed in the CNS (75), at comparable levels as glutamate and GABA receptors (74, 76). On the other hand, CB2R was originally thought to be restricted to immune and hematopoietic cells (77, 78), but more precise localization tools have subsequently allowed to assess its expression in other systems, including the nervous system (79) and the inner ear (80). CB2R expression in the healthy brain is in fact hundreds of times less than for CB1R but is strongly upregulated under pathological conditions (81). Localization, splice variants, and physiology of CBRs appear to be highly species-dependent (73), thus complicating result comparisons between animal and human studies.

CB1R neuronal effects are well known and have been extensively covered in several exhaustive reviews (49, 82, 83). Glial responses are less completely characterized but appear important especially in the presence of neuroinflammation (84), where EC tone is elevated (85). Neuroinflammation is a protective brain defense response that can however degenerate into a chronical state involved in the pathophysiology of several neurological and psychiatric disorders (86).

In neurons (Figure 1Aa), the classical EC effect is retrograde inhibition mediated by presynaptic neuronal CB1Rs and postsynaptically produced 2-AG: CB1R activation inhibits the release of the presynaptic transmitter (22), causing short-term DSE on excitatory neurons, or DSI on inhibitory neurons (87). This mechanism has been dissected in the DCN molecular layer, where glutamatergic parallel fibers carrying non-auditory signals contact fusiform cells and glycinergic cartwheel cells (which in turn provide feedforward inhibition to fusiform cells) (88). Fusiform cell output is shaped by plasticity in the molecular layer circuits, which collectively generate “negative images” of expected sounds to be attenuated at fusiform apical dendrites (89). Plasticity changes in this circuit have been correlated with tinnitus onset (90, 91). Cartwheel cells release EC from their dendrites upon stimulation, thus inducing DSE at parallel fibers (92), whereas fusiform cells do not; therefore, activation of cartwheel cells depresses its parallel fiber input, gradually reducing their feedforward inhibition (93). In fusiform cells, ECs are involved in acetylcholine-induced plasticity changes at parallel fiber synapses (94) which have been correlated with tinnitus (95). Prolonged exposure to high doses of salicylate (a well-known tinnitus inducer) increases EC release in the DCN, thus changing molecular layer plasticity (96). Unfortunately, cannabinoid modulation of this circuit has not yielded effective tinnitus treatments [see discussion in (97)].

For AEA, on the other hand, the biosynthetic enzyme NAPE-PLD is both pre- and postsynaptic, and the catabolic enzyme FAAH-1 is predominantly postsynaptic (1). Postsynaptic production of AEA produces a “tonic” retrograde inhibition at some synapses, which is shut down by neuronal inactivity through upregulation of FAAH1 (98); presynaptic production feeds instead into an anterograde mechanism. In addition, in the hippocampus, NAPE-PLD is localized in intracellular membrane cisternae of axonal Ca^2+^ stores (99) and AEA may act as an intracellular messenger by activating TRPV1 intracellular binding site.

Like neurons, glial cells can synthesize ECs in response to physiological or pathological stimuli (100, 101). In astrocytes, more than 70% of CB1Rs are found at perivascular endfeet, and EC activation has been found to modulate brain energy consumption (102) through EC effects on astrocyte mitochondria (103). At synapses, astrocytes express both DAGLα and MAGL and may display Ca^2+^-dependent EC release, which modulates synaptic response (104); conversely, astrocytic CB1R activation may induce Ca^2+^-dependent release of Glu (105), ATP, or D-serine (106) in response to synaptic EC. Astrocyte EC effects have been found to be involved in the regulation of sleep in the PPT (107) and in the regulation of circadian rhythms in the suprachiasmatic nucleus (108). These latter effects may be relevant for tinnitus given its association with sleep disturbances (109) and its circadian modulation (110).

Neuroinflammation is a brain reaction aimed at counteracting acute damage, restoring the homeostasis and limiting brain parenchyma injury, and includes microglial activation, reactive astrogliosis, production of inflammatory mediators, BBB breakdown, and subsequent brain infiltration of circulating immune cells (111). Neuroinflammation dysregulation may turn microglia and astrocytes in uncontrolled sources of inflammatory mediators, which may worsen damage progression.

A growing body of data suggest that EC are able to exert immunoregulatory and anti-inflammatory properties (112–114), by decreasing the production of NO, ROS/RNS, free radicals, and pro-inflammatory cytokines in activated glial cells, while switching microglia toward anti-inflammatory phenotypes (115–118). Remarkably, the increase in EC concentration and microglial CB receptors during neuroinflammation may yield a neuroprotective negative feedback mechanism aimed at limiting inflammatory responses.

The main brain source of ECs in neuroinflammatory conditions is microglia (119, 120), the resident immune cells of the CNS (121–123). Consistently with its immune role and nature, microglia express DAGL-β and (mainly) ABHD12 instead of the neuronal DAGL-α and MAGL (124), and while CB1Rs are expressed at low levels and mostly located intracellularly (120), microglia is the main CB2R-expressing cell in the brain (125). Microglial CB2R expression may increase up to 100 fold upon inflammation or tissue injury (126), and microglial Ca^2+^ increases [e.g., from P2X7 receptor activation (127)] and directly increases DAGL, thus increasing the production of 2-AG (128), which during neuroinflammation becomes 20-fold higher in microglia than in other brain cells (120). Mounting evidence suggests that the EC system might represent a promising tool to modify (micro)glial activity and profiles in order to achieve benefits for neuroinflammatory diseases (104). Indeed, CB2Rs can downregulate astrocyte and microglial cell overactivation during neuroinflammatory disorders, thus protecting them (129); selective depletion of MAGL in astrocytes attenuates LPS-induced neuroinflammation [(130), and CB2R upregulation and activation of EC signaling pathways have been associated with a restoration of tissue homeostasis in neuroinflammatory conditions (118, 131).

Brain CB2Rs have been less studied than CB1Rs (79), mainly due to the delay in the availability of sensitive genetic and molecular tools (126, 132, 133). In the CNS, CB2Rs are chiefly expressed on microglia (134, 135), and to some extent on astrocytes, oligodendrocytes, progenitor neural cells, and neurons (136–138); neuronal CB2R is mainly postsynaptic, differently from CB1R (137). In human, the brain only expresses one CB2R isoform (CB2RA) whereas a second one (CB2RB) is expressed in the immune system (139); rats express two additional isoforms (CB2RC and CB2RD) present neither in mice nor in humans (126), and their CB2R expression is lower and with a different distribution from mice (140). Lack of CB2R brain expression was incorrectly inferred by methods only evidencing non-brain isoforms or with insufficient sensitivity (126).

Microglial actions range from protection against damaging signals altering CNS homeostasis through phagocytosis, release of proinflammatory cytokines, and recruitment of circulating immune cells [reviewed in (141)], to controlling neuronal proliferation and differentiation [through selective neuronal phagocytosis and release of neurotrophic and neurotoxic factors reviewed in (142)], to modulating neuronal plasticity and memory [through neurotrophin release and selective synaptic pruning reviewed in (141, 142)]. In order to fulfill all these tasks, microglia are extremely plastic cells that readily change their phenotypes on demand; microglial phenotypes, previously crammed into an M1–M2 gradient to fit a classical macrophage activation model (143), are now recognized to be much more diverse (144) and influenced by the brain region (145), species (146), age (147), gender (148), and physiopathological state (149). In particular, neurodegenerative diseases appear to associate with specific microglial phenotypes which release pro-inflammatory mediators, as well as contributing to prolonged oxidative stress, leading to chronic neuroinflammation, which in turn drives neurodegeneration (141, 150, 151).

As regards hearing loss, which is a risk factor for tinnitus, chronic inflammation is seen as a major player in presbycusis [reviewed in (152)] and has been found to be associated with poorer hearing in a population-based cross-sectional study (153). Moreover, in mice, microglial ablation and TNF-alpha antagonism (154) both decrease tinnitus signs, and TNF-alpha KO mice are resilient to noise trauma-induced tinnitus (154). In human, gene polymorphisms in both TNF-alpha (155) and IL-6 (156) have been found to increase tinnitus risk in an elderly population with a history of occupational noise exposure. Neuroinflammation (and its dysregulation) appears therefore as a promising candidate mechanism for tinnitus susceptibility, and its modulation by cannabinoids may provide novel therapeutic targets. A caveat regarding neuroinflammation as a target is the complexity emerging from single-cell studies (157), which could underlie a heterogeneity similar to that observed in most multifactorial inflammatory disorders [e.g., rheumatoid arthritis (158), Menière's disease (159), and IBD (160)].

Besides neurons and glial cells, neuroinflammation involves cells of the immune system, where EC cellular mechanisms differ from neuronal ones. Cannabinoid immunomodulatory effects are complex but appear to be largely mediated through CB2Rs, whose expression on immune cells is usually higher than that of CB1R (161, 162). Moreover, nonclassical cannabinoid targets such as TRP channels (53) and PPARs (15) are well-known as key regulators of the immune response (163–165). It is interesting that EC responses in the cochlea (see below) appear more similar to those observed in the immune system than in the nervous system.

In human immune cells, CB2R is expressed most in B cells, followed by NK cells, monocytes, neutrophils, and finally T cells (134, 166, 167). Peripheral blood T cells, monocytes, and dendritic cells only express intracellular CB2R (168), whereas naïve peripheral blood B cells also express these receptors on the cell surface and lose it upon activation (169). Intracellular CB2Rs in immune cells have been associated with Ca^2+^ release from stores (3).

CB2R activation in immune cells regulates all three major MAPKs (12) and decreases DNA binding for various nuclear factors (170), which results in the downregulation of critical immunoregulatory genes including IL-2 (171, 172). Overall, these major signaling networks play important roles in CB2R-mediated effects on immune cell functions including migration, proliferation, differentiation, apoptosis, and cytokine production (28). Generally, effects of the EC system on immune cells appear directed toward an anti-inflammatory action, although the context-dependent action of cannabinoids may support different responses in different cell types and states (62, 173–176).

As regards neuroinflammatory responses, a major player is the Toll-like receptor (TLR) system (177). TLRs are able to recognize pathogen-associated and damage-associated molecular patterns (PAMPs and DAMPs), and several of their effects appear to be counteracted by ECs [especially through CB2R-related mechanisms (11)]. Since cochlear damage has also been found to induce TLR4-responses (178), similar protective effects could be expected on the cochlea.

### Cannabinoids and Tinnitus

Cannabinoids have been considered as potential treatment for tinnitus percept and/or distress, and with the legalization of light cannabis (L.242/2016 as regards Italy), several tinnitus sufferers are turning to it as a possible DIY remedy. Interest in cannabinoids as possible treatment for tinnitus has been motivated by several reasons. Early models of tinnitus stressed its similarities with neuropathic pain (179) and with epilepsy (180), both of which can be modulated by cannabinoids (181, 182).

The association between tinnitus and marijuana use in humans has been studied with contrasting results. In one study on health problems related to illicit drug use from the NSDUH database (*n* = 29,195) (183), tinnitus did not show any association to marijuana use (whereas an association was found with hallucinogens and inhalants); in a second, cross-sectional study on the NHANES database (*n* = 2,705) (184), a correlation was found between tinnitus and cannabis use, although not between cannabis use frequency and tinnitus severity, and the authors concluded that it was not possible to differentiate between causal association (cannabis use increases tinnitus prevalence), reverse causal association (tinnitus sufferers use more cannabis than non-sufferers), and association due to external common cause (i.e., anxiety, which increases both tinnitus risk and cannabis use).

Animal studies [reviewed in (97)] suggest that cannabinoids do not reduce, and may even favor, tinnitus percept. Similarly, tinnitus in humans has been sporadically observed in association with abuse of synthetic cannabinoid mixtures (185, 186).

**Figure 2:**
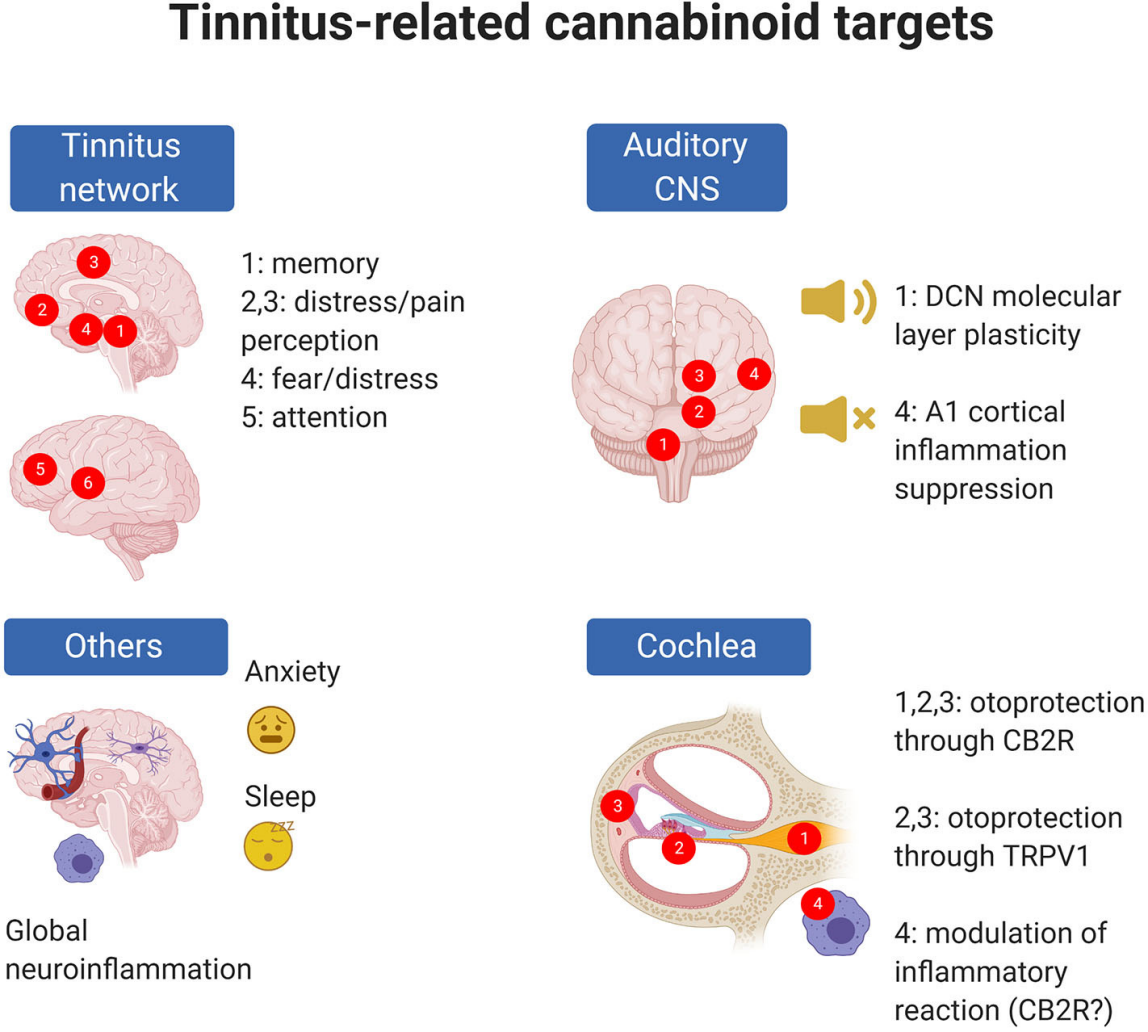
Tinnitus-related EC targets are present in the cochlea and central auditory system but also in CNS circuits altered in tinnitus; moreover, ECs may target phenomena which are known to be associated with tinnitus risk (e.g., anxiety) even though precise cellular mechanisms are uncertain. In panel “Tinnitus network,” numbers indicate as follows: 1: parahippocampal cortex; 2: ventromedial prefrontal cortex; 3: cingulate cortex; 4: amygdala; 5: dorsolateral prefrontal cortex; 6: insula (from 273). In panel “Auditory CNS” numbers indicate as follow: 1: cochlear nuclei; 2: auditory pons and midbrain; 3: medial geniculate body; 4: auditory cortex. In panel “Cochlea,” numbers indicate as follows: 1: spiral ganglion; 2: organ of Corti; 3: stria vascularis; 4: cochlear macrophages. Created with Biorender.

These seemingly contradictory results arise from two inherent complexities in the problem under study. First, the responses to cannabinoids (even for the same compound mixtures) strongly depend on drug formulation, administration route, and concentration. Second (Figure 2), tinnitus can result from many different mechanisms which are often hard to identify.

As regards the first complexity, it is important to stress that isolated and characterized phytocannabinoids, present in *Cannabis sativa* L. and a few other plant species (187, 188), include about 120 molecules (189), the most studied of which are Δ9-THC, mainly responsible for cannabis psychoactive effects (55), and cannabidiol (CBD), the major non-psychotropic component (190).

After the explanation of the structure–activity relationships in the Δ9-THC series (191, 192), a large and heterogeneous array of cannabimimetic compounds (Supplementary Tables 1, 2) have been synthesized (193, 194) including cannabinoid receptor agonists and antagonists (195), as well as drugs acting on EC metabolism (18, 196). Although, for several of them, dangerous health effects and strong potential for abuse and addiction greatly limit therapeutic use (197–199), several synthetic and phyto-cannabinoids are currently under clinical evaluation for different pathological conditions (see Table 1).

**Table 1:** Major clinical trials based on pharmacological treatment targeting the endocannabinoid system (updated to July 21, 2020).

**Drug**	**Pharmacology**	**Phase**	**Conditions**	Completion date (*estimated date for ongoing studies)	**National clinical trials (NCT) number**
AZD1940	CB_1_/CB_2_ non-selective agonist	1 2	Back pain Pain	November 2008 May 2008	NCT00689780, NCT00659490
Org 28611	CB_1_/CB_2_ non-selective agonist	2	Pain	August 2007	NCT00782951
SAB378	CB_1_/CB_2_ non-selective agonist	2	Pain	January 2010	NCT00723918
APD-371	CB_2_ selective agonist	2 2	Abdominal Pain, Crohn disease Abdominal pain	September 2018 *February 2022	NCT03155945 NCT04043455
GW-842,166X	CB_2_ selective agonist	**1** **2**	Inflammatory pain Pain	July 2007 May 2009	NCT00511524 NCT00444769
Lenabasum	CB_2_ selective agonist	**2** **3** **3** **2** **2** **2** **2**	Chronic inflammation	*August 2020 *December 2021 March 2020 December 2016 *July 2023 *December 2020 *December 2021	NCT03451045 NCT03813160 NCT03398837 NCT02465450 NCT02466243 NCT03093402 NCT02465437
Cannabidiol (Epidiolex)	See cannabidiol section for details	1,2 3 3 3 1 3 3 3 1	Seizures	May 2016 June 2017 February 2019 *February 2022 August 2019 March 2016 May 2016 *January 2021 June 2019	NCT02324673 NCT02318602 NCT02544763 NCT02544750 NCT02700412 NCT02224690 NCT02224560 NCT03808935 NCT02286986
		**1** **1** **1** **1** **1** **1,2** **2** **3** **2** **2** **3** **3** **3** **3** **3** **3** **3** **3** **3** **3** **1** **2** **3** 2 4	Pain Chronic pain Neuropathic pain	December 2019 December 2020 *December 2021 *December 2021 January 2015 *December 2021 December 2019 August 2002 *December 2020 January 2010 January 2016 September 2006 January 2005 November 2014 July 2015 December 2015 August 2002 September 2002 September 2008 March 2004 *September 2022 November 2018 December 2004 July 2020 June 2020	NCT04193631 NCT03215940 NCT04044729 NCT04030442 NCT01893424 NCT02751359 NCT04088929 NCT01606176 NCT03099005 NCT00530764 NCT01337089 NCT00675948 NCT01606202 NCT01361607 NCT01262651 NCT01424566 NCT01604265 NCT01606189 NCT00391079 NCT00674609 NCT03679949 NCT03763851 NCT01606137 NCT04195269 NCT03891264
		**2** **2** **2**	Anxiety	*August 2021 *February 2021 *March 2022	NCT02548559 NCT04267679 NCT04286594
		**2** **1** **2** **2** **2** **2,3** **3**		*January 2021 *November 2021 *July 2021 November 2017 *June 2021 February 2017 *October 2020	NCT04086342 NCT04075435 NCT03948074 NCT02818777 NCT03582137 NCT02283281 NCT03549819
ABX-1431	MAGL inhibitor	1 1 1 1 1 2	Pain Neurodegerative disorders	March 2018 July 2018 May 2019 October 2017 July 2018 January 2020	NCT02929264 NCT03138421 NCT03447756 NCT03058562 NCT03138421 NCT03625453
ASP8477	FAAH inhibitor	2	Neuropathic pain	February 2015	NCT02065349
JNJ-42165279	FAAH inhibitor	1 1 2 2 2	Anxiety	July 2014 August 2014 August 2018 February 2019 *March 2022	NCT02169973 NCT01826786 NCT02432703 NCT02498392 NCT03664232
PF-04457845	FAAH inhibitor	1 1 2 2 2 2 2	Pain	July 2009 March 2017 May 2010 March 2015 June 2020 June 2020 *December 2022	NCT00836082 NCT02134080 NCT00981357 NCT02216097 NCT01618656 NCT01665573 NCT03386487
SSR411298	FAAH inhibitor	2 2	Pain	February 2010 February 2012	NCT00822744 NCT01439919
V158866	FAAH inhibitor	1 2	Neuropathic pain	July 2011 July 2015	NCT01634529 NCT01748695

Cannabinoid bioavailability varies significantly by their formulation and route of administration (200, 201) and is also affected by poorly controllable factors such as subjective inhalation characteristics (200, 202, 203) or hepatic first-pass metabolism (202, 204–206). This is particularly relevant because the expansion of legal use of cannabinoids, for medical and nonmedical purposes, has substantially increased the types of commercially available preparations (207).

Second, besides the intrinsic complexities of cannabinoid pharmacology, the main problem in attempting a pharmacological approach to tinnitus is the lack of a clear unifying causative hypothesis for this condition (208, 209). Current models of tinnitus include (1) a peripheral trigger [which is assumed to be reduced or altered cochlear input (210), even if transient (211) or “hidden” [but see (212)], or possibly a somatosensory trigger (210, 213)]; (2) an aberrant compensatory response in the brainstem [most likely more complex than a simple “gain increase” (91, 210, 214) as was initially postulated to compensate for reduced input (215)]; and (3) a reconfiguration of cortical pathways including auditory, attentional, salience-related, and emotion-processing networks [which is thought to be necessary for the tinnitus percept to emerge to consciousness (216, 217)]. Given the absence of a causative hypothesis for tinnitus, in this review we will consider cannabinoid effects linked to both tinnitus and its main risk factors such as hearing loss or anxiety.

In animal models, tinnitus may be induced by noise trauma or ototoxic drugs such as salicylate (218). In humans, tinnitus is associated with several risk factors such as hearing loss, head trauma, and endocrine and immune dysregulation (208); however, the association between risk factors and tinnitus is far from linear. For example, although hearing loss is the main risk factor for tinnitus, it is not always accompanied by it, and tinnitus may be present without hearing loss (208). Non-auditory brain circuits also play important roles: in particular, tinnitus shows comorbidity with anxiety and depression (208, 219) and chronic tinnitus is associated with changes in attentional, memory, and limbic circuits (220, 221). The hypothesis explaining the involvement of non-auditory circuits includes a misdirection of attention which stays anomalously focused on the tinnitus percept (216), the involvement of limbic circuits encoding distress (220, 221) and the “replaying” of phantom sounds from memory in the absence of real percepts (220, 221).

At each of the levels thought to be associated with tinnitus onset and chronicization there are both well-known and potential cannabinoid targets. EC mechanisms have been found in the auditory brainstem, and particularly in the DCN, which is thought to be a major site of tinnitus onset (91, 222, 223). These neuronal, CB1R-based mechanisms (see previous section for a discussion of DCN effects) were considered very promising for a cannabinoid-based tinnitus treatment; unfortunately, animal studies displayed no effects, or even tinnitus increase, upon treatment [see discussion in (97)].

In addition to these targets, however, several other EC mechanisms (mainly related to inflammation) are present in the auditory system and in other CNS regions important for tinnitus (Figure 2). A protective EC mechanism is present in the cochlea (224, 225). Moreover, animal studies show inflammatory responses in the auditory cortex after tinnitus induction (154, 226), and inflammatory responses in the cochlea (154, 227, 228) and cochlear nuclei (229–232) after tinnitus-inducing treatments. Neuroinflammation may uncover novel EC-related therapeutic strategies, given the well-known anti-inflammatory effect of several cannabinoid drugs and pathways (see previous section).

In the auditory system, EC receptors and biosynthetic enzymes have been observed in several species and at several levels, and EC system modulation affects hearing at various levels. Moreover, several immune components and mechanisms known to be affected by EC modulation are also present in the auditory system, both peripheral and central. In the mammalian auditory system, EC system components or effects have been found in the cochlea (80), cochlear nuclei (93, 96, 233), MNTB (234), inferior colliculus (235, 236), and auditory cortex (237).

The hearing phenotypes of knockout mice for CB1R (238) and ABHD12 (239) have been characterized. In CB1R KO mice, high-frequency hearing is reduced but gap detection is improved, suggesting a change in auditory processing (238) or attentional modulation of perception, since in humans, chronic cannabis use is associated with attention-modulated deficit in PPI (240). Of relevance for tinnitus, CB1R KO mice also exhibit increased anxiety responses (241).

ABHD12 KO mice (239) and human ABHD12 nonsense mutations (242) display progressive hearing loss within PHARC syndrome. The absence of functional ABHD12 removes a catabolic pathway for 2-AG (see Figure 1); although the causative link between mutation and phenotype is still missing, a pro-inflammatory phenotype displaying microglial activation is observed (243), consistent with the expression of ABHD12 in both resting and activated microglia (242). Moreover, in the ABHD12 KO mouse the AA-related lipidome displays significant brain region-dependent changes (239) and macrophages increase LPS-induced cytokine production (244). On the other hand, the selective block of ABHD12 in adult mice does not induce hearing loss, suggesting developmental effects (245).

KO mice for CB2R (246) and other EC system components (239) are available, but their hearing has not been characterized; CB2R KO mice, on the other hand, display significant memory alterations (247).

In the cochlea, CB1R mRNA has been detected, and it decreases upon tinnitus-inducing salicylate treatment (248). However, the role of CB1Rs in the cochlea is still uncertain. On the other hand, CB2Rs have been found in rodent hair cells and pillar and Deiters' cells, spiral ganglion and nerve, and stria vascularis basal cells (224), and their expression increases upon cisplatin administration (80). Cisplatin is known to be strongly ototoxic by inducing cochlear inflammation (249), and CB2R block or knockdown makes the cochlea more sensitive to cisplatin ototoxicity (224): moreover, treatment with the CB2R antagonist AM630 is in itself proinflammatory, suggesting the presence of a cytoprotective EC tone in the cochlea (224). In addition, EC protective role in the cochlea has been found to involve TRPV1 activation: TRPV1 channels are expressed in hair cells (especially toward the apical pole), pillar, and Deiters' cells and in the marginal cells of the stria vascularis (250). The TRPV1 agonist capsaicin increases cochlear CB2R expression, and a CB2R-dependent mechanism induces the activation of STAT3; on the other hand, cisplatin induces the activation of proapoptotic factor STAT1 (225). The protective effect of capsaicin, which transiently induces STAT1 and TTS (225), is most likely due to the strong desensitization it induces on TRPV1 channels after a transient activation, similar to its effect in pain treatment (251).

CB1Rs are present in both ventral (VCN) and dorsal (DCN) cochlear nuclei of the rat; in the VCN, their role is unclear, but their expression decreases upon salicylate treatment, which induces tinnitus (233). In the DCN, salicylate does not change CB1R expression (233) but alters EC response on cartwheel cells (96). It is interesting to note the presence of CB1R (252) and CB2R (253) in the IV ventricle choroid plexus, especially because CB2R promote neural stem cell proliferation (254) and neurogenesis was observed in cochlear nuclei after deafferentation (255). In both man (256) and rat (257), there is a variable direct contact between the DCN surface and branches of the choroid plexus, where ECs released in the DCN molecular layer (92) could reach the plexus, possibly modulating its immune gate function (258).

As regards cortical effects important for tinnitus treatment, it is well known that anxiety (181) and attention (259) are strongly affected by cannabinoids. A point to be remembered is that, although cannabis use is associated with an acute anti-anxiety effect (260), chronic cannabis use may dramatically worsen anxiety (261, 262), thus exacerbating tinnitus severity. The anxiety-inducing effect of cannabis is correlated with its Δ9-THC content, and Δ9-THC alone may induce anxiety and paranoia (263); on the other hand, CBD appears to have opposite effects on anxiety (264) and is currently under clinical evaluation for the treatment of anxiety, psychosis, and posttraumatic stress disorder (190, 265, 266).

These data show that cannabinoid effects of possible relevance for tinnitus are very diverse and include anti-inflammatory, protective reactions and selective circuit modulation of “auditory context.” Since the anti-inflammatory route is starting to be explored as a possible therapeutic target in hearing loss (152) and tinnitus (154), interest has been raised for cannabinoids as a treatment option, and in particular for CBD, owing to its good toxicological profile in humans and lack of psychotropic effects. The recent availability of CBD preparations underlies anecdotal use reports by tinnitus patients; however, no controlled human studies have been performed yet.

Cannabidiol (CBD) is currently under clinical evaluation for the treatment of pain, anxiety, depression, sleep disorders, PTSD, headaches, and seizures (see Supplementary Table 1), all conditions which display analogies or associations with tinnitus (97, 179, 208). Despite such a wide spectrum of potentially interesting pharmacological properties, the practical effects of CBD on tinnitus are still underexplored.

Indeed, as of today the only study using CBD investigated the effects of a THC-CBD 1:1 mixture on noise trauma-induced tinnitus in the rat, showing no effects of daily treatment on tinnitus animals, and actually suggesting that cannabinoids might favor tinnitus onset, since treatment increased the fraction of animals showing tinnitus signs (267). These results agree with the effects of synthetic CB1R agonists (WIN55, 212-2, CP55,940, and ACEA) which have been tested in animal models of salicylate-induced tinnitus, with negative results [rat: (268); guinea pig: (269)]. It has to be remembered, however, that co-administered CBD and THC interact in a very complex way, and cannabinoid mixtures exert effects which may be very different from the simple combination of the effects of each drug *per se* (270). One example is CB1R activation in the cerebral cortex and hippocampus, associated with effects on cognition and memory (271): in this model, CBD is able to counteract THC-induced memory impairment (272).

In general, the pharmacodynamic of CBD appears particularly complex, with over 65 identified molecular targets, and different mechanisms proposed to explain its actions (190, 273, 274). Here we summarize only the CBD targets which may bear relevance for tinnitus.

On CB1R/CB2R, CBD has a very low affinity (in the μM range) and shows little agonist activity; on the other hand, it seems to antagonize CB1/CB2 synthetic agonist action with KB values in the nM range (275). It has been suggested that CBD acts as negative allosteric modulator of CB1R and as antagonist/inverse agonist of CB2R (276); in addition, it may indirectly affect CBR function by inhibiting FAAH activity, thus increasing endogenous anandamide levels (277, 278). For example, CBD neuroprotective effect after cerebral hypoxia–ischemia in immature pigs involves CB2R activation (279) and may be therefore due to EC increase rather than to a direct receptor effect.

Besides these effects, CBD acts as antagonist/inverse agonist of GPCR3, GPCR6, GPCR18, and GPCR55 (33, 280) and modulates serotonergic transmission acting as an allosteric agonist of 5HT1A receptor, a partial agonist of 5HT2A, and an allosteric inhibitor of 5HT3A (281–283). CBD protective effects on a BBB permeability model (284) required PPARγ and 5HT1A and were independent of CBRs. Similarly, CBD anti-depressant and anxiolytic effects also appear independent from CB2R (285) and linked to 5HT1A activation.

In the μM range, CBD may also activate adenosine A1 (286) and A2A receptors (287), activate glycine α1 (288) and α3 receptors (289), inhibit α7 nicotinic acetylcholine receptors (290), and allosterically modulate μ and δ opioid receptors (half maximal inhibition was observed at ~10 μM) (291). As a caveat, since CBD concentrations > 20 μM are unlikely to be attained *in vivo* (292), not all the described CBD pharmacological activities are likely to be physiologically meaningful.

Modulation of α7 nAChRs may be relevant for tinnitus since these receptors are expressed in cortical and hippocampal neurons and affect cognition and memory [reviewed in (293)]; moreover, these receptors are also expressed in microglia (294) and macrophages (295) and are involved in the vagal-mediated cholinergic anti-inflammatory response signaling through the JAK2/STAT3 pathway, decreasing levels of pro-inflammatory cytokines, such as TNF-α, IL-1β, and IL-6 and increasing levels of anti-inflammatory cytokines such as IL-10 (295–298).

Finally, CBD may affect several ion channels including voltage-dependent Na channels (299), T-type Ca channels (300), and TRPV1 and TRPV2 channels (301). In particular, CBD can act on TRPV-1, exhibiting an action similar to capsaicin, both *in vitro* (302) and in an animal model of acute inflammation (303). This is relevant since capsaicin is able to exert protective effects on cochlear inflammatory damage (225), and therefore, CBD may exert similar otoprotective actions.

## Conclusions

1. Cannabinoids are involved in neural processing in the healthy auditory system, in protective reaction to auditory damage, and in most non-auditory circuits known to be associated with tinnitus.
2. Given the availability of a large number of drugs with a wide spectrum of different effects on the EC system, it appears possible that some of them may reduce tinnitus percept or risk factors rather than increase them, similar to what is seen, e.g., for anxiety (where EC-targeting drugs may either worsen or ameliorate it).
3. EC modulation of neuroinflammatory responses in the auditory system, in particular by CBD, which is neuroprotective, is anti-inflammatory, undergoes clinical trial as an anxiolytic, and acts on pathways involved in cochlear damage protection, may represent a novel pharmacological approach to hearing loss and tinnitus, although more data are necessary (especially on humans) to assess the therapeutic value of this or other EC drugs.

## Author Contributions

All authors listed have made a substantial, direct and intellectual contribution to the work, and approved it for publication. PP, RP, and PE contributed auditory expertise. AMT, FM, and MC neuroimmunological expertise. PE and CB pharmacological and clinical expertise.

## Conflict of Interest

The authors declare that the research was conducted in the absence of any commercial or financial relationships that could be construed as a potential conflict of interest.

## Abbreviations

2-AG: 2-arachidonoylglycerol
ABHD4, ABHD6, ABHD12: αβ-hydrolase domain 4,6,12
ACEA: arachidonyl-2'-chloroethylamide
AEA: N-arachidonoylethanolamide (anandamide)
BDNF: brain-derived nerve factor
BNST: bed nucleus of the stria terminalis
CBR; CB1R; CB2R: cannabinoid receptor, type 1, type 2
CBD: cannabidiol
COX-2: cyclooxygenase type 2
CREB: cAMP response element binding protein
DAG: diacylglycerol
DAGL: DAG lipase
DCN: dorsal cochlear nucleus
DSE: Depolarization-induced suppression of excitation
DSI: Depolarization-induced suppression of inhibition
EC: endocannabinoid
EMT: endocannabinoid membrane transporter
ERK: Extracellular signal-Regulated Kinase
FAAH: fatty acid amide hydrolase
GDE1: glycerophosphodiesterase 1
GPCR: G-protein-coupled receptor
HPA: hypothalamic-pituitary-adrenal
MAGL: monoacylglycerol lipase
NAPE: N-arachidoylphosphatidyletanolamine
NAT: N-acyltransferase
NR3C1: glucocorticoid receptor
PHARC: Polyneuropathy, Hearing loss, Ataxia, Retinitis pigmentosa, and Cataracts
PLA2: phospholipase A2
PLD: phospholipase D
PPAR: Peroxisome proliferator-activated receptors
PUFA: polyunsaturated fatty acids
SPM: specialized pro-resolving mediators
TRP: Transient receptor potential
Δ9-THC: Δ9-tetrahydrocannabinol
VCN: ventral cochlear nucleus.
