# Supplementary material for: Cannabinoids, Inner Ear, Hearing, and Tinnitus: A Neuroimmunological Perspective
Source: Front Neurol. 2020 Nov 23;11:505995. doi: 10.3389/fneur.2020.505995 (PMC7719758; doi:10.3389/fneur.2020.505995)
Supplement: Supplementary file 1 [file Table_1.docx]

**Table S1.** Synthetic and naturally occurring ligands for endocannabinoid receptors. Abbreviations: nd, no data; aka, also known as.

| **Ligand** | **CB_1_** | | | **CB_2_** | | | **Other targets** | **References** |
| --- | --- | --- | --- | --- | --- | --- | --- | --- |
|  | **K_i_**  (nM)  ***mean***  *(range or ±SEM)* | **EC_50_**  (nM)  ***mean***  *(range or ±SEM)* | **Action** | **K_i_**  (nM)  ***mean***  *(range or ±SEM)* | **EC_50_**  (nM)  ***mean***  *(range or ±SEM)* | **Action** |  |  |
| **Non selective CB_1_ and CB_2_ agonists** | | | | | | | | |
| (–)-Δ^9^-THC  aka dronabinol | 5.05–80.3 | nd | partial agonist | 3.13–75.3 | nd | partial agonist | Na^+^ channels, glycine receptors, 5-HT_3A_ receptors, noradrenaline transporter, dopamine transporter, 5-HT transporter, GPR55, GPR18, TPRV2, TRPA1, TRPM8, μ– and δ-opioid receptors | Anavi-Goffer et al., 2012; Jordt et al., 2004; Kapur et al., 2009; McHugh et al., 2012; Oz, 2006; Pertwee, 2008, 2004, 1988; Qin et al., 2008; Ryberg et al., 2007 |
| 2-arachidonoylglycerol | 58.3; 472 | nd | agonist | 145; 1,400 | nd | agonist | GPR55, Monoacylglycerol lipase, αβ-Hydrolase 6 | Ghafouri et al., 2004; Navia-Paldanius et al., 2012; Pertwee, 2005; Ryberg et al., 2007 |
| AM-2201 | 1.0 | nd | agonist | 2.6 | nd | agonist | nd | Makriyannis and Deng, 2001 |
| AM7499 | 2.51 | 2.5 | agonist | 31.62 | 0.3 | agonist | nd | S. Kulkarni et al., 2016 |
| anandamide | 61–543 | nd | partial agonist | 279–1,940 | nd | partial agonist | N-type Ca^2+^ channels, T-type Ca^2+^ channels, Na^+^ channels, Ca^2+^-activated K^+^ channels, other types of voltage-gated K^+^ channels, α7 nAch channels, glycine receptors, NMDA, 5-HT_3A_, TRPV4, TRPV1, GPR55, GPR18, TRPM8 | McHugh et al., 2012; Oz, 2006; Pertwee, 2005, 2004, 1988; Ryberg et al., 2009 |
| AZD1940  aka ART27.13 | 11.75 | nd | agonist  (peripherally restricted) | 0.87 | nd | agonist  (peripherally restricted) | nd | Schou et al., 2013 |
| BAY 38-7271  aka KN 38-7271 | 0.46-1.85 | nd | agonist | 5.96 | nd | agonist | nd | Mauler et al., 2002 |
| BMS-202 | nd | 100 | agonist | nd | 125.9 | agonist | programmed cell death 1 ligand 1 | Banister et al., 2015; Guzik et al., 2017 |
| cannabinol | 211 | 120 | partial agonist | 126 | 261 | nd | TRPV2 | Felder et al., 1995; Qin et al., 2008; Rhee et al., 1997; Showalter et al., 1996; Zygmunt et al., 2002 |
| CP-55,940 | 0.5-5.0 | nd | agonist | 0.69-2.8 | nd | agonist | 5-HT_3A_, TRPV1, GPR55 | Kapur et al., 2009; Oz, 2006; Pertwee, 2004, 1999 |
| HU-210 | 0.06–0.73 | nd | agonist | 0.17–0.52 | nd | agonist | 5-HT_2_ receptors, TRPV1, GPR55, glycine receptors | Cheer et al., 1999; Oz, 2006; Pertwee, 2005, 2004; Ryberg et al., 2007; Yang et al., 2008 |
| JWH-007 | 9.50 | nd | agonist | 2.94 | nd | agonist | nd | Aung et al., 2000 |
| JWH-018  aka AM-678 | 9.00±5.00 | nd | agonist | 2.94±2.65 | nd | agonist | nd | Aung et al., 2000 |
| JWH-073 | 8.90±1.80 | nd | partial agonist | 38.0±24.0 | nd | partial agonist | nd | Aung et al., 2000 |
| MDMB-Fubinaca  aka FUB-MDMB | 0.1 | 0.06 | agonist | 0.13 | 0.76 | agonist | nd | Schoeder et al., 2018 |
| nabilone | 2.19±0.89 | nd | agonist | 1.8±0.42 | nd | agonist | nd | Gareau et al., 1996 |
| O-1057 | 4.4 | nd | agonist | 11.2 | nd | agonist | nd | Pertwee, 2000 |
| Org 28611  aka SCH-900,111 | 1.26 | 25.12 | agonist | 1.58 | nd | agonist | nd | Adam et al., 2010 |
| SAB378  aka CB-13 | 15±5 | nd | agonist  (peripherally restricted) | 98±7.6 | nd | agonist (peripherally restricted) | nd | Dziadulewicz et al., 2007 |
| WIN55212-2 | 1.89–123 | nd | agonist | 0.28–16.2 | nd | agonist | 5-HT_3A_, TRPV1, glycine receptors | Pertwee, 2005, 2004; Yang et al., 2008 |
| **Selective CB_1_ agonists/positive allosteric modulator** | | | | | | | | |
| 2-arachidonyl glyceryl ether  aka Noladin ether | 21.2 | nd | agonist | ˃3,000 | nd | nd | GPR55 | Pertwee, 2005; Ryberg et al., 2007 |
| AM11542 | 0.1 | nd | agonist | nd | nd | nd | nd | Hua et al., 2017 |
| AM841 | 1.26 | nd | agonist | nd | nd | nd | nd | Hua et al., 2017 |
| arachidonyl-2-chloroethylamide | 1.4; 5.29 | nd | agonist | 195; ˃2,000 | nd | nd | nd | Hillard et al., 1999; Lin et al., 1998; Pertwee, 2005 |
| arachidonylcyclopropylamide | 2.2±0.4 | nd | agonist | 700±10 | nd | nd | GPR18 | Hillard et al., 1999; McHugh et al., 2012 |
| JD5037 | 0.35 | 1.5 | inverse agonist  (peripherally-restricted) | nd | nd | nd | nd | Tam et al., 2012 |
| LDK1229 | 220 |  | inverse agonist | nd | nd | nd | nd | Mahmoud et al., 2015 |
| levonantradol  aka CP 50,556-1 | 2.3-4.9 | nd | agonist | nd | nd | nd | nd | Breivogel and Childers, 2000 |
| MK-9470 | nd | 0.7 | inverse agonist | nd | nd | nd | nd | Burns et al., 2007 |
| O-1812 | 3.4 | nd | agonist | 3870 | nd | nd | nd | Di Marzo et al., 2001 |
| Org27569 | nd | 158.49 | positive allosteric modulator | nd | nd | nd | nd | Piscitelli et al., 2012 |
| R-(+)-methanandamide | 17.9–28.3 | nd | nd | 815–868 | nd | nd | nd | Pertwee, 2005 |
| taranabant  aka MK-0364 | 0.13; 0.27 | 2.8 | inverse agonist | 170; 310 | nd | nd | nd | Fong et al., 2007 |
| ZCZ011 | nd | 125.9-501.2 | positive allosteric modulator | nd | nd | nd | nd | Ignatowska-Jankowska et al., 2015 |
| α-/β-amyrin | 0.133 | nd | agonist | 1,989 | nd | nd | nd | Chicca et al., 2012 |
| **Selective CB_2_ agonists** | | | | | | | | |
| AM1241 | 280 | nd | nd | 3.4 | nd | agonist | nd | Pertwee, 2005 |
| AM1710 | 398.1 | nd | agonist | 6.3 | nd | agonist | nd | Khanolkar et al., 2007 |
| APD-371  aka olorinab | nd | >10,000 | agonist | 19.4 | 6.2 | agonist | nd | Han et al., 2017 |
| GW-405,833  aka L-768,242 | 2,043 | nd | nd | 14 | 0.65 | partial agonist | nd | Gallant et al., 1996; Huffman and Marriott, 2008 |
| GW-842,166X | nd | ˃30,000 | nd | nd | 63 | agonist | nd | Giblin et al., 2009, 2007 |
| HU-308 | ˃10,000 | nd | nd | 22.7 | nd | agonist | glycine receptors | Pertwee, 2005; Yang et al., 2008 |
| JTE-907 | 2,370 | nd | nd | 35.9 | nd | inverse agonist | nd | Pertwee, 2005 |
| JWH-015 | 383 | nd | agonist | 13.8 | nd | agonist | 5-HT_3A_, GPR55 | Pertwee, 2005, 2004; Ryberg et al., 2007 |
| JWH-133 | 677 | nd | nd | 3.4 | nd | agonist | nd | Pertwee, 2005 |
| L-759,633 | 1,043;  15,850 | nd | nd | 6.4;  20 | nd | agonist | nd | Gareau et al., 1996; Ross et al., 1999 |
| L-759,656 | 529->20,000 | nd | nd | 35-19.4 | nd | agonist | nd | Gareau et al., 1996; Huffman et al., 2002, 1999; Ross et al., 1999 |
| lenabasum  aka JBT-101 | 628 | nd | agonist | 51 | 13.4 | agonist | nd | Tepper et al., 2014 |
| S-777469 | nd | nd | nd | nd | 36 | agonist | nd | Odan et al., 2012 |
| Sch.336 | 1,000 | 199.5 | inverse agonist | 0.4-2 | 2 | inverse agonist | nd | Lavey et al., 2005; Lunn et al., 2005 |
| UR-144  aka TMCP-018 or KM-X1 | 150 | 421 | nd | 1.8 | 72 | agonist | nd | Frost et al., 2010 |
| XL-001 | nd | nd | nd | 0.5 | nd | inverse agonist | nd | Zhou et al., 2018 |
| **Selective CB_1_ competitive antagonists/negative allosteric modulator** | | | | | | | | |
| AM251 | 7.5 | nd | antagonist | 2,290 | nd | nd | GPR55, μ-opioid receptors | Ruoxi Lan et al., 1999; Ryberg et al., 2009; Seely et al., 2012 |
| AM281 | 12 | nd | antagonist | 4,200 | nd | nd | GPR55 | Henstridge et al., 2010; R Lan et al., 1999 |
| AM6538 | 5 | nd | antagonist | nd | nd | nd | nd | Hua et al., 2016 |
| AM6545 | 3.2 | nd | antagonist | nd | nd | nd | nd | Bowles et al., 2015 |
| cannabidiol | nd | 4,350 | negative allosteric modulator | 2,860 | nd | nd | GPR18, GPR55, TPRV2, TPRV3, TRPM8 | De Petrocellis et al., 2012; Laprairie et al., 2015; McHugh et al., 2012; Pertwee et al., 2010; Qin et al., 2008; Ryberg et al., 2009; Showalter et al., 1996 |
| compound 70 | 64.3 | nd | antagonist | nd | nd | nd | nd | Sharma et al., 2015 |
| dietressa | nd | nd | antagonist antibody | nd | nd | nd | nd | Barchukov et al., 2015 |
| drinabant  aka AVE-1625 | 0.16-0.44 | 25±4 | antagonist | nd | ˃ 10,000 | nd | nd | Herling et al., 2007 |
| GAT-100 | nd | 19.95-398.11 | negative allosteric modulator  (irreversible) | nd | nd | nd | nd | P. M. Kulkarni et al., 2016; Laprairie et al., 2016 |
| ibipinabant  aka SLV-319 | 7.8±1.4 | nd | antagonist | 7,943±126 | antagonist | nd | nd | Lange et al., 2004 |
| LY320135 | 141 | nd | antagonist | 14,900 | nd | nd | nd | Felder et al., 1998 |
| MJ08 | 25.4 | 78.6 | antagonist/inverse agonist | nd | nd | nd | nd | Chen et al., 2011 |
| NESS 0327 | 0.35 | nd | antagonist | 21 | ˃ 10,000 | nd | nd | Ruiu et al., 2003 |
| otenabant  aka CP-945,598 | 0.7, 0.12 | nd | antagonist/inverse agonist | 7,600 | nd | nd | nd | Griffith et al., 2009; Hadcock et al., 2010 |
| pepcan-12 | 5.01-7.94 | nd | negative allosteric modulator | nd | nd | nd | nd | Bauer et al., 2012 |
| PSNCBAM-1 | nd | 45; 209 | negative allosteric modulator | nd | ˃10,000 | nd | nd | German et al., 2014; Horswill et al., 2007 |
| rimonabant  aka SR141716A | 1.8–12.3 | nd | antagonist/inverse agonist | 514–13,200 | nd | nd | GPR55 | Brown et al., 2011; Rinaldi-Carmona et al., 1994 |
| surinabant  aka SR147778 | 0.56; 3.5 | nd | antagonist | 400 | nd | agonist | nd | Rinaldi-Carmona et al., 2004 |
| TM-38837 | nd | 7.94 | antagonist | nd | 630.9 | antagonist | nd | Hung et al., 2010 |
| VCE-004.3 | 2511.89 | nd | antagonist | 199.53 | nd | agonist | nd | del Rio et al., 2018 |
| **Selective CB_2_ competitive antagonists** | | | | | | | | |
| AM10257 | nd | nd | nd | 0.08 | nd | antagonist | nd | Li et al., 2019 |
| AM-630 | 5152 | ˃10,000 | nd | 31.2 | 76.6 | antagonist | nd | Ross et al., 1999 |
| SR144528 | 50.3–˃10,000 | nd | nd | 0.28–5.6 | nd | antagonist | nd | Pertwee, 2005 |

**References**

Adam, J.M., Cairns, J., Caulfield, W., Cowley, P., Cumming, I., Easson, M., Edwards, D., Ferguson, M., Goodwin, R., Jeremiah, F., Kiyoi, T., Mistry, A., Moir, E., Morphy, R., Tierney, J., York, M., Baker, J., Cottney, J.E., Houghton, A.K., Westwood, P.J., Walker, G., 2010. Design, synthesis, and structure-activity relationships of indole-3-carboxamides as novel water soluble cannabinoid CB1 receptor agonists †. Med. Chem. Commun. 1, 54–60. https://doi.org/10.1039/c0md00022a

Anavi-Goffer, S., Baillie, G., Irving, A.J., Gertsch, J., Greig, I.R., Pertwee, R.G., Ross, R.A., 2012. Modulation of L-α-lysophosphatidylinositol/GPR55 mitogen-activated protein kinase (MAPK) signaling by cannabinoids. J. Biol. Chem. 287, 91–104. https://doi.org/10.1074/jbc.M111.296020

Aung, M.M., Griffin, G., Huffman, J.W., Wu, M.-J., Keel, C., Yang, B., Showalter, V.M., Abood, M.E., Martin, B.R., 2000. Influence of the N-1 alkyl chain length of cannabimimetic indoles upon CB1 and CB2 receptor binding. Drug Alcohol Depend. 60, 133–140. https://doi.org/10.1016/S0376-8716(99)00152-0

Banister, S.D., Stuart, J., Kevin, R.C., Edington, A., Longworth, M., Wilkinson, S.M., Beinat, C., Buchanan, A.S., Hibbs, D.E., Glass, M., Connor, M., McGregor, I.S., Kassiou, M., 2015. Effects of Bioisosteric Fluorine in Synthetic Cannabinoid Designer Drugs JWH-018, AM-2201, UR-144, XLR-11, PB-22, 5F-PB-22, APICA, and STS-135. ACS Chem. Neurosci. 6, 1445–1458. https://doi.org/10.1021/acschemneuro.5b00107

Barchukov, V. V., Zhavbert, E.S., Dugina, Y.L., Epstein, O.I., 2015. The Use of Release-Active Antibody-Based Preparations for Vertigo Prevention in Adults. Bull. Exp. Biol. Med. 160, 61–63. https://doi.org/10.1007/s10517-015-3098-z

Bauer, M., Chicca, A., Tamborrini, M., Eisen, D., Lerner, R., Lutz, B., Poetz, O., Pluschke, G., Gertsch, J., 2012. Identification and Quantification of a New Family of Peptide Endocannabinoids (Pepcans) Showing Negative Allosteric Modulation at CB1 Receptors. J. Biol. Chem. 287, 36944–36967. https://doi.org/10.1074/jbc.M112.382481

Bowles, N.P., Karatsoreos, I.N., Li, X., Vemuri, V.K., Wood, J.-A., Li, Z., Tamashiro, K.L.K., Schwartz, G.J., Makriyannis, A.M., Kunos, G., Hillard, C.J., McEwen, B.S., Hill, M.N., 2015. A peripheral endocannabinoid mechanism contributes to glucocorticoid-mediated metabolic syndrome. Proc. Natl. Acad. Sci. 112, 285–290. https://doi.org/10.1073/pnas.1421420112

Breivogel, C.S., Childers, S.R., 2000. Cannabinoid agonist signal transduction in rat brain: comparison of cannabinoid agonists in receptor binding, G-protein activation, and adenylyl cyclase inhibition. J. Pharmacol. Exp. Ther. 295, 328–36.

Brown, A.J., Daniels, D.A., Kassim, M., Brown, S., Haslam, C.P., Terrell, V.R., Brown, J., Nichols, P.L., Staton, P.C., Wise, A., Dowell, S.J., 2011. Pharmacology of GPR55 in yeast and identification of GSK494581A as a mixed-activity glycine transporter subtype 1 inhibitor and GPR55 agonist. J. Pharmacol. Exp. Ther. 337, 236–46. https://doi.org/10.1124/jpet.110.172650

Burns, H.D., Van Laere, K., Sanabria-Bohorquez, S., Hamill, T.G., Bormans, G., Eng, W. -s., Gibson, R., Ryan, C., Connolly, B., Patel, S., Krause, S., Vanko, A., Van Hecken, A., Dupont, P., De Lepeleire, I., Rothenberg, P., Stoch, S.A., Cote, J., Hagmann, W.K., Jewell, J.P., Lin, L.S., Liu, P., Goulet, M.T., Gottesdiener, K., Wagner, J.A., de Hoon, J., Mortelmans, L., Fong, T.M., Hargreaves, R.J., 2007. [18F]MK-9470, a positron emission tomography (PET) tracer for in vivo human PET brain imaging of the cannabinoid-1 receptor. Proc. Natl. Acad. Sci. 104, 9800–9805. https://doi.org/10.1073/pnas.0703472104

Cheer, J.F., Cadogan, A.-K., Marsden, C.A., Fone, K.C.F., Kendall, D.A., 1999. Modification of 5-HT 2 receptor mediated behaviour in the rat by oleamide and the role of cannabinoid receptors, Neuropharmacology.

Chen, W., Xu, C., Liu, H., Long, L., Zhang, W., Zheng, Z., Xie, Y., Wang, L., Li, S., 2011. Novel selective cannabinoid CB(1) receptor antagonist MJ08 with potent in vivo bioactivity and inverse agonistic effects. Acta Pharmacol. Sin. 32, 1148–58. https://doi.org/10.1038/aps.2011.80

Chicca, A., Marazzi, J., Gertsch, J., 2012. The antinociceptive triterpene β-amyrin inhibits 2-arachidonoylglycerol (2-AG) hydrolysis without directly targeting cannabinoid receptors. Br. J. Pharmacol. 167, 1596–608. https://doi.org/10.1111/j.1476-5381.2012.02059.x

De Petrocellis, L., Orlando, P., Moriello, A.S., Aviello, G., Stott, C., Izzo, A.A., Di Marzo, V., 2012. Cannabinoid actions at TRPV channels: effects on TRPV3 and TRPV4 and their potential relevance to gastrointestinal inflammation. Acta Physiol. (Oxf). 204, 255–266. https://doi.org/10.1111/j.1748-1716.2011.02338.x

del Rio, C., Cantarero, I., Palomares, B., Gómez-Cañas, M., Fernández-Ruiz, J., Pavicic, C., García-Martín, A., Luz Bellido, M., Ortega-Castro, R., Pérez-Sánchez, C., López-Pedrera, C., Appendino, G., Calzado, M.A., Muñoz, E., 2018. VCE-004.3, a cannabidiol aminoquinone derivative, prevents bleomycin-induced skin fibrosis and inflammation through PPARγ- and CB2 receptor-dependent pathways. Br. J. Pharmacol. 175, 3813–3831. https://doi.org/10.1111/bph.14450

Di Marzo, V., Bisogno, T., De Petrocellis, L., Brandi, I., Jefferson, R.G., Winckler, R.L., Davis, J.B., Dasse, O., Mahadevan, A., Razdan, R.K., Martin, B.R., 2001. Highly Selective CB1 Cannabinoid Receptor Ligands and Novel CB1/VR1 Vanilloid Receptor “Hybrid” Ligands. Biochem. Biophys. Res. Commun. 281, 444–451. https://doi.org/10.1006/BBRC.2001.4354

Dziadulewicz, E.K., Bevan, S.J., Brain, C.T., Coote, P.R., Culshaw, A.J., Davis, A.J., Edwards, L.J., Fisher, A.J., Fox, A.J., Gentry, C., Groarke, A., Hart, T.W., Huber, W., James, I.F., Kesingland, A., Vecchia, L. La, Loong, Y., Lyothier, I., Mcnair, K., O’farrell, C., Peacock, M., Portmann, R., Schopfer, U., Yaqoob, M., Zadrobilek, J., 2007. Naphthalen-1-yl-(4-pentyloxynaphthalen-1-yl)methanone: A Potent, Orally Bioavailable Human CB 1 /CB 2 Dual Agonist with Antihyperalgesic Properties and Restricted Central Nervous System Penetration. https://doi.org/10.1021/jm070317a

Felder, C.C., Joyce, K.E., Briley, E.M., Glass, M., Mackie, K.P., Fahey, K.J., Cullinan, G.J., Hunden, D.C., Johnson, D.W., Chaney, M.O., Koppel, G.A., Brownstein, M., 1998. LY320135, a novel cannabinoid CB1 receptor antagonist, unmasks coupling of the CB1 receptor to stimulation of cAMP accumulation. J. Pharmacol. Exp. Ther. 284, 291–7.

Felder, C.C., Joyce, K.E., Briley, E.M., Mansouri, J., Mackie, K., Blond, O., Lai, Y., Ma, A.L., Mitchell, R.L., 1995. Comparison of the pharmacology and signal transduction of the human cannabinoid CB1 and CB2 receptors. Mol. Pharmacol. 48, 443–50.

Fong, T.M., Guan, X.-M., Marsh, D.J., Shen, C.-P., Stribling, D.S., Rosko, K.M., Lao, J., Yu, H., Feng, Y., Xiao, J.C., Van der Ploeg, L.H.T., Goulet, M.T., Hagmann, W.K., Lin, L.S., Lanza, T.J., Jewell, J.P., Liu, P., Shah, S.K., Qi, H., Tong, X., Wang, J., Xu, S.S., Francis, B., Strack, A.M., MacIntyre, D.E., Shearman, L.P., 2007. Antiobesity Efficacy of a Novel Cannabinoid-1 Receptor Inverse Agonist, N-[(1S,2S)-3-(4-Chlorophenyl)-2-(3-cyanophenyl)-1-methylpropyl]-2-methyl-2- propanamide (MK-0364), in Rodents. J. Pharmacol. Exp. Ther. 321, 1013–1022. https://doi.org/10.1124/jpet.106.118737

Frost, J.M., Dart, M.J., Tietje, K.R., Garrison, T.R., Grayson, G.K., Daza, A. V., El-Kouhen, O.F., Yao, B.B., Hsieh, G.C., Pai, M., Zhu, C.Z., Chandran, P., Meyer, M.D., 2010. Indol-3-ylcycloalkyl Ketones: Effects of N1 Substituted Indole Side Chain Variations on CB 2 Cannabinoid Receptor Activity. J. Med. Chem. 53, 295–315. https://doi.org/10.1021/jm901214q

Gallant, M., Dufresne, C., Gareau, Y., Guay, D., Leblanc, Y., Prasit, P., Rochette, C., Sawyer, N., Slipetz, D.M., Tremblay, N., Metters, K.M., Labelle, M., 1996. New class of potent ligands for the human peripheral cannabinoid receptor. Bioorg. Med. Chem. Lett. 6, 2263–2268. https://doi.org/10.1016/0960-894X(96)00426-X

Gareau, Y., Dufresne, C., Gallant, M., Rochette, C., Sawyer, N., Slipetz, D.M., Tremblay, N., Weech, P.K., Metters, K.M., Labelle, M., 1996. Structure activity relationships of tetrahydrocannabinol analogues on human cannabinoid receptors. Bioorg. Med. Chem. Lett. 6, 189–194. https://doi.org/10.1016/0960-894X(95)00573-C

German, N., Decker, A.M., Gilmour, B.P., Gay, E.A., Wiley, J.L., Thomas, B.F., Zhang, Y., 2014. Diarylureas as allosteric modulators of the cannabinoid CB1 receptor: structure-activity relationship studies on 1-(4-chlorophenyl)-3-{3-[6-(pyrrolidin-1-yl)pyridin-2-yl]phenyl}urea (PSNCBAM-1). J. Med. Chem. 57, 7758–69. https://doi.org/10.1021/jm501042u

Ghafouri, N., Tiger, G., Razdan, R.K., Mahadevan, A., Pertwee, R.G., Martin, B.R., Fowler, C.J., 2004. Inhibition of monoacylglycerol lipase and fatty acid amide hydrolase by analogues of 2-arachidonoylglycerol. Br. J. Pharmacol. 143, 774–784. https://doi.org/10.1038/sj.bjp.0705948

Giblin, G.M.P., Billinton, A., Briggs, M., Brown, A.J., Chessell, I.P., Clayton, N.M., Eatherton, A.J., Goldsmith, P., Haslam, C., Johnson, M.R., Mitchell, W.L., Naylor, A., Perboni, A., Slingsby, B.P., Wilson, A.W., 2009. Discovery of 1-[4-(3-Chlorophenylamino)-1-methyl-1H-pyrrolo[3,2-c]pyridin-7-yl]-1-morpholin-4-ylmethanone (GSK554418A), a Brain Penetrant 5-Azaindole CB2 Agonist for the Treatment of Chronic Pain. J. Med. Chem. 52, 5785–5788. https://doi.org/10.1021/jm9009857

Giblin, G.M.P., O’Shaughnessy, C.T., Naylor, A., Mitchell, W.L., Eatherton, A.J., Slingsby, B.P., Rawlings, D.A., Goldsmith, P., Brown, A.J., Haslam, C.P., Clayton, N.M., Wilson, A.W., Chessell, I.P., Wittington, A.R., Green, R., 2007. Discovery of 2-[(2,4-dichlorophenyl)amino]-N-[(tetrahydro- 2H-pyran-4-yl)methyl]-4-(trifluoromethyl)- 5-pyrimidinecarboxamide, a selective CB2 receptor agonist for the treatment of inflammatory pain. J. Med. Chem. 50, 2597–600. https://doi.org/10.1021/jm061195+

Griffith, D.A., Hadcock, J.R., Black, S.C., Iredale, P.A., Carpino, P.A., DaSilva-Jardine, P., Day, R., DiBrino, J., Dow, R.L., Landis, M.S., O’Connor, R.E., Scott, D.O., 2009. Discovery of 1-[9-(4-Chlorophenyl)-8-(2-chlorophenyl)-9 H-purin-6-yl]-4-ethylaminopiperidine-4-carboxylic Acid Amide Hydrochloride (CP-945,598), a Novel, Potent, and Selective Cannabinoid Type 1 Receptor Antagonist. J. Med. Chem. 52, 234–237. https://doi.org/10.1021/jm8012932

Guzik, K., Zak, K.M., Grudnik, P., Magiera, K., Musielak, B., Törner, R., Skalniak, L., Dömling, A., Dubin, G., Holak, T.A., 2017. Small-Molecule Inhibitors of the Programmed Cell Death-1/Programmed Death-Ligand 1 (PD-1/PD-L1) Interaction via Transiently Induced Protein States and Dimerization of PD-L1. J. Med. Chem. 60, 5857–5867. https://doi.org/10.1021/acs.jmedchem.7b00293

Hadcock, J.R., Griffith, D.A., Iredale, P.A., Carpino, P.A., Dow, R.L., Black, S.C., O’Connor, R., Gautreau, D., Lizano, J.S., Ward, K., Hargrove, D.M., Kelly-Sullivan, D., Scott, D.O., 2010. In vitro and in vivo pharmacology of CP-945,598, a potent and selective cannabinoid CB1 receptor antagonist for the management of obesity. Biochem. Biophys. Res. Commun. 394, 366–371. https://doi.org/10.1016/j.bbrc.2010.03.015

Han, S., Thoresen, L., Jung, J.-K., Zhu, X., Thatte, J., Solomon, M., Gaidarov, I., Unett, D.J., Yoon, W.H., Barden, J., Sadeque, A., Usmani, A., Chen, C., Semple, G., Grottick, A.J., Al-Shamma, H., Christopher, R., Jones, R.M., 2017. Discovery of APD371: Identification of a Highly Potent and Selective CB 2 Agonist for the Treatment of Chronic Pain. ACS Med. Chem. Lett. 8, 1309–1313. https://doi.org/10.1021/acsmedchemlett.7b00396

Henstridge, C.M., Balenga, N.A., Schröder, R., Kargl, J.K., Platzer, W., Martini, L., Arthur, S., Penman, J., Whistler, J.L., Kostenis, E., Waldhoer, M., Irving, A.J., 2010. GPR55 ligands promote receptor coupling to multiple signalling pathways. Br. J. Pharmacol. 160, 604–614. https://doi.org/10.1111/j.1476-5381.2009.00625.x

Herling, A.W., Gossel, M., Haschke, G., Stengelin, S., Kuhlmann, J., Müller, G., Schmoll, D., Kramer, W., 2007. CB1 receptor antagonist AVE1625 affects primarily metabolic parameters independently of reduced food intake in Wistar rats. Am. J. Physiol. Metab. 293, E826–E832. https://doi.org/10.1152/ajpendo.00264.2007

Hillard, C.J., Manna, S., Greenberg, M.J., DiCamelli, R., Ross, R.A., Stevenson, L.A., Murphy, V., Pertwee, R.G., Campbell, W.B., 1999. Synthesis and characterization of potent and selective agonists of the neuronal cannabinoid receptor (CB1). J. Pharmacol. Exp. Ther. 289, 1427–33.

Horswill, J.G., Bali, U., Shaaban, S., Keily, J.F., Jeevaratnam, P., Babbs, A.J., Reynet, C., Wong Kai In, P., 2007. PSNCBAM-1, a novel allosteric antagonist at cannabinoid CB1 receptors with hypophagic effects in rats. Br. J. Pharmacol. 152, 805–14. https://doi.org/10.1038/sj.bjp.0707347

Hua, T., Vemuri, K., Nikas, S.P., Laprairie, R.B., Wu, Y., Qu, L., Pu, M., Korde, A., Jiang, S., Ho, J.-H., Han, G.W., Ding, K., Li, X., Liu, H., Hanson, M.A., Zhao, S., Bohn, L.M., Makriyannis, A., Stevens, R.C., Liu, Z.-J., 2017. Crystal structures of agonist-bound human cannabinoid receptor CB1. Nature 547, 468–471. https://doi.org/10.1038/nature23272

Hua, T., Vemuri, K., Pu, M., Qu, L., Han, G.W., Wu, Y., Zhao, S., Shui, W., Li, S., Korde, A., Laprairie, R.B., Stahl, E.L., Ho, J.-H., Zvonok, N., Zhou, H., Kufareva, I., Wu, B., Zhao, Q., Hanson, M.A., Bohn, L.M., Makriyannis, A., Stevens, R.C., Liu, Z.-J., 2016. Crystal Structure of the Human Cannabinoid Receptor CB 1. Cell 167, 750-762.e14. https://doi.org/10.1016/j.cell.2016.10.004

Huffman, J., Marriott, K.-S., 2008. Recent Advances in the Development of Selective Ligands for the Cannabinoid CB2 Receptor. Curr. Top. Med. Chem. 8, 187–204. https://doi.org/10.2174/156802608783498014

Huffman, J.W., Bushell, S.M., Miller, J.R.., Wiley, J.L., Martin, B.R., 2002. 1-Methoxy-, 1-deoxy-11-hydroxy- and 11-Hydroxy-1-methoxy-Δ8-tetrahydrocannabinols: new selective ligands for the CB2 receptor. Bioorg. Med. Chem. 10, 4119–4129. https://doi.org/10.1016/S0968-0896(02)00331-0

Huffman, J.W., Liddle, J., Yu, S., Aung, M.M., Abood, M.E., Wiley, J.L., Martin, B.R., 1999. 3-(1’,1’-Dimethylbutyl)-1-deoxy-delta8-THC and related compounds: synthesis of selective ligands for the CB2 receptor. Bioorg. Med. Chem. 7, 2905–14.

Hung, M.-S., Chang, C.-P., Li, T.-C., Yeh, T.-K., Song, J.-S., Lin, Y., Wu, C.-H., Kuo, P.-C., Amancha, P.K., Wong, Y.-C., Hsiao, W.-C., Chao, Y.-S., Shia, K.-S., 2010. Discovery of 1-(2,4-Dichlorophenyl)-4-ethyl-5-(5-(2-(4-(trifluoromethyl)phenyl)ethynyl)thiophen-2-yl)-N-(piperidin-1-yl)-1H-pyrazole-3-carboxamide as a Potential Peripheral Cannabinoid-1 Receptor Inverse Agonist. ChemMedChem 5, 1439–1443. https://doi.org/10.1002/cmdc.201000246

Ignatowska-Jankowska, B.M., Baillie, G.L., Kinsey, S., Crowe, M., Ghosh, S., Owens, R.A., Damaj, I.M., Poklis, J., Wiley, J.L., Zanda, M., Zanato, C., Greig, I.R., Lichtman, A.H., Ross, R.A., 2015. A Cannabinoid CB1 Receptor-Positive Allosteric Modulator Reduces Neuropathic Pain in the Mouse with No Psychoactive Effects. Neuropsychopharmacology 40, 2948–2959. https://doi.org/10.1038/npp.2015.148

Jordt, S.-E., Bautista, D.M., Chuang, H., McKemy, D.D., Zygmunt, P.M., Högestätt, E.D., Meng, I.D., Julius, D., 2004. Mustard oils and cannabinoids excite sensory nerve fibres through the TRP channel ANKTM1. Nature 427, 260–265. https://doi.org/10.1038/nature02282

Kapur, A., Zhao, P., Sharir, H., Bai, Y., Caron, M.G., Barak, L.S., Abood, M.E., 2009. Atypical Responsiveness of the Orphan Receptor GPR55 to Cannabinoid Ligands. J. Biol. Chem. 284, 29817–29827. https://doi.org/10.1074/jbc.M109.050187

Khanolkar, A.D., Lu, D., Ibrahim, M., Duclos, Jr., R.I., Thakur, G.A., Malan, Jr., T.P., Porreca, F., Veerappan, V., Tian, X., George, C., Parrish, D.A., Papahatjis, D.P., Makriyannis, A., 2007. Cannabilactones: A Novel Class of CB2 Selective Agonists with Peripheral Analgesic Activity. J. Med. Chem. 50, 6493–6500. https://doi.org/10.1021/jm070441u

Kulkarni, P.M., Kulkarni, A.R., Korde, A., Tichkule, R.B., Laprairie, R.B., Denovan-Wright, E.M., Zhou, H., Janero, D.R., Zvonok, N., Makriyannis, A., Cascio, M.G., Pertwee, R.G., Thakur, G.A., 2016. Novel Electrophilic and Photoaffinity Covalent Probes for Mapping the Cannabinoid 1 Receptor Allosteric Site(s). J. Med. Chem. 59, 44–60. https://doi.org/10.1021/acs.jmedchem.5b01303

Kulkarni, S., Nikas, S.P., Sharma, R., Jiang, S., Paronis, C.A., Leonard, M.Z., Zhang, B., Honrao, C., Mallipeddi, S., Raghav, J.G., Benchama, O., Järbe, T.U.C., Bergman, J., Makriyannis, A., 2016. Novel C-Ring-Hydroxy-Substituted Controlled Deactivation Cannabinergic Analogues. J. Med. Chem. 59, 6903–6919. https://doi.org/10.1021/acs.jmedchem.6b00717

Lan, R, Gatley, J., Lu, Q., Fan, P., Fernando, S.R., Volkow, N.D., Pertwee, R., Makriyannis, A., 1999. Design and synthesis of the CB1 selective cannabinoid antagonist AM281: a potential human SPECT ligand. AAPS PharmSci 1, E4.

Lan, Ruoxi, Liu, Q., Fan, P., Lin, S., Fernando, S.R., McCallion, D., Pertwee, R., Makriyannis, A., 1999. Structure−Activity Relationships of Pyrazole Derivatives as Cannabinoid Receptor Antagonists. J. Med. Chem. 42, 769–776. https://doi.org/10.1021/jm980363y

Lange, J.H.M., Coolen, H.K.A.C., van Stuivenberg, H.H., Dijksman, J.A.R., Herremans, A.H.J., Ronken, E., Keizer, H.G., Tipker, K., McCreary, A.C., Veerman, W., Wals, H.C., Stork, B., Verveer, P.C., den Hartog, A.P., de Jong, N.M.J., Adolfs, T.J.P., Hoogendoorn, J., Kruse, C.G., 2004. Synthesis, Biological Properties, and Molecular Modeling Investigations of Novel 3,4-Diarylpyrazolines as Potent and Selective CB (1) Cannabinoid Receptor Antagonists. J. Med. Chem. 47, 627–643. https://doi.org/10.1021/jm031019q

Laprairie, R.B., Bagher, A.M., Kelly, M.E.M., Denovan-Wright, E.M., 2015. Cannabidiol is a negative allosteric modulator of the cannabinoid CB 1 receptor. Br. J. Pharmacol. 172, 4790–4805. https://doi.org/10.1111/bph.13250

Laprairie, R.B., Kulkarni, A.R., Kulkarni, P.M., Hurst, D.P., Lynch, D., Reggio, P.H., Janero, D.R., Pertwee, R.G., Stevenson, L.A., Kelly, M.E.M., Denovan-Wright, E.M., Thakur, G.A., 2016. Mapping Cannabinoid 1 Receptor Allosteric Site(s): Critical Molecular Determinant and Signaling Profile of GAT100, a Novel, Potent, and Irreversibly Binding Probe. ACS Chem. Neurosci. 7, 776–798. https://doi.org/10.1021/acschemneuro.6b00041

Lavey, B.J., Kozlowski, J.A., Hipkin, R.W., Gonsiorek, W., Lundell, D.J., Piwinski, J.J., Narula, S., Lunn, C.A., 2005. Triaryl bis-sulfones as a new class of cannabinoid CB2 receptor inhibitors: identification of a lead and initial SAR studies. Bioorg. Med. Chem. Lett. 15, 783–786. https://doi.org/10.1016/j.bmcl.2004.11.007

Li, X., Hua, T., Vemuri, K., Ho, J.-H., Wu, Y., Wu, L., Popov, P., Benchama, O., Zvonok, N., Locke, K., Qu, L., Han, G.W., Iyer, M.R., Cinar, R., Coffey, N.J., Wang, J., Wu, M., Katritch, V., Zhao, S., Kunos, G., Bohn, L.M., Makriyannis, A., Stevens, R.C., Liu, Z.-J., 2019. Crystal Structure of the Human Cannabinoid Receptor CB2. Cell 176, 459-467.e13. https://doi.org/10.1016/J.CELL.2018.12.011

Lin, S., Khanolkar, A.D., Fan, P., Goutopoulos, A., Qin, C., Papahadjis, D., Makriyannis, A., 1998. Novel Analogues of Arachidonylethanolamide (Anandamide): Affinities for the CB1 and CB2 Cannabinoid Receptors and Metabolic Stability. J. Med. Chem. 41, 5353–5361. https://doi.org/10.1021/jm970257g

Lunn, C.A., Fine, J.S., Rojas-Triana, A., Jackson, J. V, Fan, X., Kung, T.T., Gonsiorek, W., Schwarz, M.A., Lavey, B., Kozlowski, J.A., Narula, S.K., Lundell, D.J., Hipkin, R.W., Bober, L.A., 2005. A Novel Cannabinoid Peripheral Cannabinoid Receptor-Selective Inverse Agonist Blocks Leukocyte Recruitment in Vivo. J. Pharmacol. Exp. Ther. 316, 780–788. https://doi.org/10.1124/jpet.105.093500

Mahmoud, M.M., Olszewska, T., Liu, H., Shore, D.M., Hurst, D.P., Reggio, P.H., Lu, D., Kendall, D.A., 2015. (4-(Bis(4-fluorophenyl)methyl)piperazin-1-yl)(cyclohexyl)methanone hydrochloride (LDK1229): a new cannabinoid CB1 receptor inverse agonist from the class of benzhydryl piperazine analogs. Mol. Pharmacol. 87, 197–206. https://doi.org/10.1124/mol.114.095471

Makriyannis, A., Deng, H., 2001. Cannabimimetic indole derivatives.

Mauler, F., Mittendorf, J., Horváth, E., De Vry, J., 2002. Characterization of the diarylether sulfonylester (-)-(R)-3-(2-hydroxymethylindanyl-4-oxy)phenyl-4,4,4-trifluoro-1-sulfonate (BAY 38-7271) as a potent cannabinoid receptor agonist with neuroprotective properties. J. Pharmacol. Exp. Ther. 302, 359–68.

McHugh, D., Page, J., Dunn, E., Bradshaw, H.B., 2012a. Δ9-Tetrahydrocannabinol and N-arachidonyl glycine are full agonists at GPR18 receptors and induce migration in human endometrial HEC-1B cells. Br. J. Pharmacol. 165, 2414–2424. https://doi.org/10.1111/j.1476-5381.2011.01497.x

McHugh, D., Page, J., Dunn, E., Bradshaw, H.B., 2012b. Δ9-Tetrahydrocannabinol and N-arachidonyl glycine are full agonists at GPR18 receptors and induce migration in human endometrial HEC-1B cells. Br. J. Pharmacol. 165, 2414–2424. https://doi.org/10.1111/j.1476-5381.2011.01497.x

Navia-Paldanius, D., Savinainen, J.R., Laitinen, J.T., 2012. Biochemical and pharmacological characterization of human α/β-hydrolase domain containing 6 (ABHD6) and 12 (ABHD12). J. Lipid Res. 53, 2413–2424. https://doi.org/10.1194/jlr.M030411

Odan, M., Ishizuka, N., Hiramatsu, Y., Inagaki, M., Hashizume, H., Fujii, Y., Mitsumori, S., Morioka, Y., Soga, M., Deguchi, M., Yasui, K., Arimura, A., 2012. Discovery of S-777469: An orally available CB2 agonist as an antipruritic agent. Bioorg. Med. Chem. Lett. 22, 2803–2806. https://doi.org/10.1016/j.bmcl.2012.02.072

Oz, M., 2006a. Receptor-independent actions of cannabinoids on cell membranes: Focus on endocannabinoids. Pharmacol. Ther. 111, 114–144. https://doi.org/10.1016/j.pharmthera.2005.09.009

Oz, M., 2006b. Receptor-independent actions of cannabinoids on cell membranes: Focus on endocannabinoids. Pharmacol. Ther. 111, 114–144. https://doi.org/https://doi.org/10.1016/j.pharmthera.2005.09.009

Pertwee, R.G., 2008. The diverse CB 1 and CB 2 receptor pharmacology of three plant cannabinoids: Δ 9 -tetrahydrocannabinol, cannabidiol and Δ 9 -tetrahydrocannabivarin. Br. J. Pharmacol. 153, 199–215. https://doi.org/10.1038/sj.bjp.0707442

Pertwee, R.G., 2005. Pharmacological Actions of Cannabinoids, HEP. Springer-Verlag.

Pertwee, R.G., 2004. Pharmacological and therapeutic targets for Δ9- tetrahydrocannabinol and cannabidiol. Euphytica 140, 73–82. https://doi.org/10.1007/s10681-004-4756-9

Pertwee, R.G., 2000. Cannabinoid receptor ligands: clinical and neuropharmacological considerations, relevant to future drug discovery and development. Expert Opin. Investig. Drugs 9, 1553–1571. https://doi.org/10.1517/13543784.9.7.1553

Pertwee, R.G., 1999. Pharmacology of cannabinoid receptor ligands. Curr. Med. Chem. 6, 635–64.

Pertwee, R.G., 1988. The central neuropharmacology of psychotropic cannabinoids. Pharmacol. Ther. 36, 189–261.

Pertwee, R.G., Howlett, A.C., Abood, M.E., Alexander, S.P.H., Di Marzo, V., Elphick, M.R., Greasley, P.J., Hansen, H.S., Kunos, G., Mackie, K., Mechoulam, R., Ross, R.A., 2010. International Union of Basic and Clinical Pharmacology. LXXIX. Cannabinoid receptors and their ligands: beyond CB₁ and CB₂. Pharmacol. Rev. 62, 588–631. https://doi.org/10.1124/pr.110.003004

Piscitelli, F., Ligresti, A., La Regina, G., Coluccia, A., Morera, L., Allarà, M., Novellino, E., Di Marzo, V., Silvestri, R., 2012. Indole-2-carboxamides as Allosteric Modulators of the Cannabinoid CB1 Receptor. J. Med. Chem. 55, 5627–5631. https://doi.org/10.1021/jm201485c

Qin, N., Neeper, M.P., Liu, Y., Hutchinson, T.L., Lubin, M.L., Flores, C.M., 2008a. TRPV2 Is Activated by Cannabidiol and Mediates CGRP Release in Cultured Rat Dorsal Root Ganglion Neurons. J. Neurosci. 28, 6231–6238. https://doi.org/10.1523/JNEUROSCI.0504-08.2008

Qin, N., Neeper, M.P., Liu, Y., Hutchinson, T.L., Lubin, M.L., Flores, C.M., 2008b. TRPV2 Is Activated by Cannabidiol and Mediates CGRP Release in Cultured Rat Dorsal Root Ganglion Neurons. J. Neurosci. 28, 6231–6238. https://doi.org/10.1523/JNEUROSCI.0504-08.2008

Rhee, M.-H., Vogel, Z., Barg, J., Bayewitch, M., Levy, R., Hanuš, L., Breuer, A., Mechoulam, R., 1997. Cannabinol Derivatives: Binding to Cannabinoid Receptors and Inhibition of Adenylylcyclase. J. Med. Chem. 40, 3228–3233. https://doi.org/10.1021/jm970126f

Rinaldi-Carmona, M., Barth, F., Congy, C., Martinez, S., Oustric, D., Pério, A., Poncelet, M., Maruani, J., Arnone, M., Finance, O., Soubrié, P., Le Fur, G., 2004. SR147778 [5-(4-Bromophenyl)-1-(2,4-dichlorophenyl)-4-ethyl-N-(1-piperidinyl)-1H-pyrazole-3-carboxamide], a New Potent and Selective Antagonist of the CB1 Cannabinoid Receptor: Biochemical and Pharmacological Characterization. J. Pharmacol. Exp. Ther. 310, 905–914. https://doi.org/10.1124/jpet.104.067884

Rinaldi-Carmona, M., Barth, F., Héaulme, M., Shire, D., Calandra, B., Congy, C., Martinez, S., Maruani, J., Néliat, G., Caput, D., Ferrara, P., Soubrié, P., Brelière, J.C., Le Fur, G., 1994. SR141716A, a potent and selective antagonist of the brain cannabinoid receptor. FEBS Lett. 350, 240–244. https://doi.org/10.1016/0014-5793(94)00773-X

Ross, R.A., Brockie, H.C., Stevenson, L.A., Murphy, V.L., Templeton, F., Makriyannis, A., Pertwee, R.G., 1999a. Agonist-inverse agonist characterization at CB 1 and CB 2 cannabinoid receptors of L759633, L759656 and AM630. Br. J. Pharmacol. 126, 665–672. https://doi.org/10.1038/sj.bjp.0702351

Ross, R.A., Brockie, H.C., Stevenson, L.A., Murphy, V.L., Templeton, F., Makriyannis, A., Pertwee, R.G., 1999b. Agonist-inverse agonist characterization at CB 1 and CB 2 cannabinoid receptors of L759633, L759656 and AM630. Br. J. Pharmacol. 126, 665–672. https://doi.org/10.1038/sj.bjp.0702351

Ruiu, S., Pinna, G.A., Marchese, G., Mussinu, J.-M., Saba, P., Tambaro, S., Casti, P., Vargiu, R., Pani, L., 2003. Synthesis and Characterization of NESS 0327: A Novel Putative Antagonist of the CB1 Cannabinoid Receptor. J. Pharmacol. Exp. Ther. 306, 363–370. https://doi.org/10.1124/jpet.103.049924

Ryberg, E., Larsson, N., Sjögren, S., Hjorth, S., Hermansson, N.-O., Leonova, J., Elebring, T., Nilsson, K., Drmota, T., Greasley, P.J., 2009a. The orphan receptor GPR55 is a novel cannabinoid receptor. Br. J. Pharmacol. 152, 1092–1101. https://doi.org/10.1038/sj.bjp.0707460

Ryberg, E., Larsson, N., Sjögren, S., Hjorth, S., Hermansson, N.-O., Leonova, J., Elebring, T., Nilsson, K., Drmota, T., Greasley, P.J., 2009b. The orphan receptor GPR55 is a novel cannabinoid receptor. Br. J. Pharmacol. 152, 1092–1101. https://doi.org/10.1038/sj.bjp.0707460

Ryberg, E., Larsson, N., Sjögren, S., Hjorth, S., Hermansson, N.-O., Leonova, J., Elebring, T., Nilsson, K., Drmota, T., Greasley, P.J., 2007. The orphan receptor GPR55 is a novel cannabinoid receptor. Br. J. Pharmacol. 152, 1092–101. https://doi.org/10.1038/sj.bjp.0707460

Schoeder, C.T., Hess, C., Madea, B., Meiler, J., Müller, C.E., 2018. Pharmacological evaluation of new constituents of “Spice”: synthetic cannabinoids based on indole, indazole, benzimidazole and carbazole scaffolds. Forensic Toxicol. 36, 385–403. https://doi.org/10.1007/s11419-018-0415-z

Schou, M., Varnäs, K., Jucaite, A., Gulyás, B., Halldin, C., Farde, L., 2013. Radiolabeling of the cannabinoid receptor agonist AZD1940 with carbon-11 and PET microdosing in non-human primate. Nucl. Med. Biol. 40, 410–414. https://doi.org/10.1016/j.nucmedbio.2012.10.011

Seely, K.A., Brents, L.K., Franks, L.N., Rajasekaran, M., Zimmerman, S.M., Fantegrossi, W.E., Prather, P.L., 2012. AM-251 and rimonabant act as direct antagonists at mu-opioid receptors: Implications for opioid/cannabinoid interaction studies. Neuropharmacology 63, 905–915. https://doi.org/10.1016/j.neuropharm.2012.06.046

Sharma, M.K., Murumkar, P.R., Barmade, M.A., Giridhar, R., Yadav, M.R., 2015. A comprehensive patents review on cannabinoid 1 receptor antagonists as antiobesity agents. Expert Opin. Ther. Pat. 25, 1093–1116. https://doi.org/10.1517/13543776.2015.1064898

Showalter, V.M., Compton, D.R., Martin, B.R., Abood, M.E., 1996a. Evaluation of binding in a transfected cell line expressing a peripheral cannabinoid receptor (CB2): identification of cannabinoid receptor subtype selective ligands. J. Pharmacol. Exp. Ther. 278, 989–99.

Showalter, V.M., Compton, D.R., Martin, B.R., Abood, M.E., 1996b. Evaluation of binding in a transfected cell line expressing a peripheral cannabinoid receptor (CB2): identification of cannabinoid receptor subtype selective ligands. J. Pharmacol. Exp. Ther. 278, 989–99.

Tam, J., Cinar, R., Liu, J., Godlewski, G., Wesley, D., Jourdan, T., Szanda, G., Mukhopadhyay, B., Chedester, L., Liow, J.-S., Innis, R.B., Cheng, K., Rice, K.C., Deschamps, J.R., Chorvat, R.J., McElroy, J.F., Kunos, G., 2012. Peripheral Cannabinoid-1 Receptor Inverse Agonism Reduces Obesity by Reversing Leptin Resistance. Cell Metab. 16, 167–179. https://doi.org/10.1016/J.CMET.2012.07.002

Tepper, M.A., Zurier, R.B., Burstein, S.H., 2014. Ultrapure ajulemic acid has improved CB2 selectivity with reduced CB1 activity. Bioorg. Med. Chem. 22, 3245–3251. https://doi.org/10.1016/j.bmc.2014.04.062

Yang, Z., Aubrey, K.R., Alroy, I., Harvey, R.J., Vandenberg, R.J., Lynch, J.W., 2008. Subunit-specific modulation of glycine receptors by cannabinoids and N-arachidonyl-glycine. Biochem. Pharmacol. 76, 1014–1023. https://doi.org/10.1016/j.bcp.2008.07.037

Zhou, L., Zhou, S., Yang, P., Tian, Y., Feng, Z., Xie, X.-Q., Liu, Y., 2018. Targeted inhibition of the type 2 cannabinoid receptor is a novel approach to reduce renal fibrosis. Kidney Int. 94, 756–772. https://doi.org/10.1016/j.kint.2018.05.023

Zygmunt, P.M., Andersson, D.A., Hogestatt, E.D., 2002. Delta 9-tetrahydrocannabinol and cannabinol activate capsaicin-sensitive sensory nerves via a CB1 and CB2 cannabinoid receptor-independent mechanism. J. Neurosci. 22, 4720–7. https://doi.org/20026430
