# Supplementary material for: Cannabinoids, Inner Ear, Hearing, and Tinnitus: A Neuroimmunological Perspective
Source: Front Neurol. 2020 Nov 23;11:505995. doi: 10.3389/fneur.2020.505995 (PMC7719758; doi:10.3389/fneur.2020.505995)
Supplement: Supplementary file 2 [file Table_2.docx]

**Table S2.** Inhibitors of endocannabinoid metabolising enzymes and transporters. Abbreviations: nd, no data; aka, also known as.

| **Ligand** | **IC_50_**  (nM)  ***mean***  *(range or ±SEM)* | **Action** | **Other targets** | **References** |
| --- | --- | --- | --- | --- |
| **Fatty acid amide hydrolase (FAAH) inhibitors** | | | | |
| AM3506 | 2.8-5.1 | irreversible | nd | Alapafuja et al., 2012 |
| AM374 | 13; 50 | irreversible | nd | Ho and Hillard, 2005 |
| Arachidonoyl serotonin (AA-5-HT) | 560-1,200 | nd | nd | Ho and Hillard, 2005 |
| Arachidonyl trifluoromethyl ketone (AACOCF_3_) | 700-4,000 | nd | nd | Ho and Hillard, 2005; Riendeau et al., 1994 |
| ARN2508 | 10 | irreversible | COX-1; COX-2 | Migliore et al., 2016 |
| ASP8477 | 1.65-57.3 | nd | nd | Watabiki et al., 2017 |
| BIA 10-2474 | 50-70 | irreversible | nd | van Esbroeck et al., 2017 |
| diazomethyl arachidonyl ketone (DAK) | 500-6,000 | irreversible | nd | Edgemond et al., 1998; Ho and Hillard, 2005 |
| dual sEH/FAAH inhibitor 11  aka compound B-11 | 7.94 | nd | nd | Kodani et al., 2018 |
| JNJ1661010 | 15.85 | reversible | nd | Karbarz et al., 2009; Keith et al., 2008 |
| JNJ40355003 | 1.3 | nd | nd | Keith et al., 2012 |
| JNJ42165279 | 70-313 | slowly reversible | nd | Keith et al., 2015 |
| JZL195 | 2 | dual FAAH and MAGL inhibition | acyloxyacyl hydrolase, MAGL | Bachovchin et al., 2014; Long et al., 2009 |
| methyl arachidonyl fluorophosphonate (MAFP) | 1-3 | irreversible | nd | Ho and Hillard, 2005 |
| MM-433593 | 100 | reversible | nd | Banijamali et al., 2014 |
| N-arachidonylglycine (NAGly) | 4,100; 7,000 | nd | nd | Huang et al., 2001; McHugh et al., 2010; Tzviel Sheskin et al., 1997 |
| O-1887 | 15±3 | nd | nd | Martin et al., 2000 |
| OL135 | 2.1 | reversible | nd | Lichtman et al., 2004 |
| palmitoylisopropylamide (PIA) | 12,900 | nd | nd | Jonsson et al., 2001 |
| PF-04457845 | 1-10 | irreversible | nd | van Esbroeck et al., 2017 |
| PF-3845 | 230±30 | irreversible | nd | Ahn et al., 2009 |
| PF750 | 6.3-501.2 | irreversible | epoxide hydrolase 2 | Ahn et al., 2007; Kodani et al., 2018 |
| phenylmethylsulfonyl fluoride (PMSF) | 290-15,000 | irreversible | nd | Ho and Hillard, 2005 |
| SSR411298 | 62.5±8.4 | reversible | nd | Griebel et al., 2018 |
| ST4070 | 9 | reversible | nd | Tuo et al., 2016 |
| URB532 | 214±79 | irreversible | nd | Kathuria et al., 2003 |
| URB597  aka KDS-4103 | 0.50±0.05 | irreversible | nd | Kathuria et al., 2003; Niforatos et al., 2007 |
| V158866 | ~60 | reversible | nd | Pawsey et al., 2016 |
| **Monoacylglycerol lipase (MAGL) inhibitors** | | | | |
| ABX-1431 | 14; 27 | irreversible | nd | Cisar et al., 2018 |
| JJKK-048 | 0.5 | irreversible | nd | Aaltonen et al., 2013 |
| JZL184 | 8 | irreversible | nd | Long et al., 2009 |
| JZL195 | 4.0 | irreversible | nd | Bachovchin et al., 2014; J. Z. Long et al., 2009 |
| KML29 | 3.2 | nd | nd | Chang et al., 2012 |
| MJN110 | 10 | nd | nd | Niphakis et al., 2013 |
| N-arachidonyl maleamide (NAM) | 140 | irreversible | nd | Saario et al., 2005 |
| URB602 | 28,000±4,000 | irreversible | nd | Hohmann et al., 2005 |
| **αβ-hydrolase domain 6 (ABHD6) inhibitors** | | | | |
| JZP-169 | 216 | irreversible | nd | Jayendra Z. Patel et al., 2015 |
| JZP-430 | 44 | irreversible | nd | Jayendra Z Patel et al., 2015 |
| KT182 | 0.24-15.1 | irreversible | nd | Hsu et al., 2013 |
| KT203 | 0.2−0.3 | irreversible  (peripherally restricted) | nd | Hsu et al., 2013 |
| LEI-106 | 800±100 | nd | nd | Janssen et al., 2014 |
| orlistat | 50 | irreversible | ABHD12 | Navia-Paldanius et al., 2012 |
| UCM710 | 2,400 | nd | nd | Marrs et al., 2011 |
| WWL 70 | 70 | nd | nd | Li et al., 2007 |
| WWL123 | 398.1 | nd | nd | Bachovchin et al., 2010 |
| **αβ-hydrolase domain 12 (ABHD12) inhibitors** | | | | |
| betulinic acid | 2,500 | nd | nd | Genet et al., 2010; Parkkari et al., 2014 |
| maslinic acid | 1,300 | nd | nd | Parkkari et al., 2014 |
| oleanolic acid | 1,600 | nd | nd | Parkkari et al., 2014 |
| ursolic acid | 1,800 | nd | nd | Parkkari et al., 2014 |
| **Endocannabinoid reuptake inhibitors** | | | | |
| (-)-5’-DMH-CBD | 14,000 | nd | nd | Bisogno et al., 2001 |
| AM1172 | 2,100-2,500 | nd | nd | Fegley et al., 2004 |
| AM404  aka N-arachidonoylaminophenol | 1-11 | nd | nd | Beltramo et al., 1997; Fowler et al., 2004; Giang et al., 1997; Piomelli et al., 1999 |
| Guineensine | 290 | nd | nd | Nicolussi et al., 2014 |
| LY-2183240 | 0.27 | nd | nd | Alexander and Cravatt, 2006; Moore et al., 2005 |
| O-2093 | 17,300 | nd | nd | Ligresti et al., 2006 |
| O-3246 | 1,400 | nd | nd | Ligresti et al., 2006 |
| OMDM-1  aka (S)-N-oleoyltyrosinol | 2.4-˃20 | nd | nd | Chu et al., 2010; Fowler et al., 2004; Ortar et al., 2003 |
| OMDM-2 | 3-˃100 | nd | nd | Fowler et al., 2004; Ortar et al., 2003 |
| RX-055 | 14-32 | irreversible | nd | Chicca et al., 2017 |
| SBFI50 | 600-3,500 | nd | nd | Kaczocha et al., 2014 |
| SBFI60 | 300- ˃10,000 | nd | nd | Kaczocha et al., 2014 |
| SBFI62 | 2,600-6,100 | nd | nd | Kaczocha et al., 2014 |
| UCM-707 | 0.8-41 | nd | nd | Fowler et al., 2004; López-Rodríguez et al., 2003 |
| VDM-11 | 6.1-11.2 | nd | nd | De Petrocellis et al., 2000; Fowler et al., 2004 |
| WOBE437 | 10-283 | nd | nd | Chicca et al., 2017; Lee et al., 2008 |

**References**

Aaltonen, N., Savinainen, J.R., Ribas, C.R., Rönkkö, J., Kuusisto, A., Korhonen, J., Navia-Paldanius, D., Häyrinen, J., Takabe, P., Käsnänen, H., Pantsar, T., Laitinen, T., Lehtonen, M., Pasonen-Seppänen, S., Poso, A., Nevalainen, T., Laitinen, J.T., 2013. Piperazine and Piperidine Triazole Ureas as Ultrapotent and Highly Selective Inhibitors of Monoacylglycerol Lipase. Chem. Biol. 20, 379–390. doi:10.1016/j.chembiol.2013.01.012

Ahn, K., Johnson, D.S., Fitzgerald, L.R., Liimatta, M., Arendse, A., Stevenson, T., Lund, E.T., Nugent, R.A., Nomanbhoy, T.K., Alexander, J.P., Cravatt, B.F., 2007. Novel Mechanistic Class of Fatty Acid Amide Hydrolase Inhibitors with Remarkable Selectivity ^†^. Biochemistry 46, 13019–13030. doi:10.1021/bi701378g

Ahn, K., Johnson, D.S., Mileni, M., Beidler, D., Long, J.Z., McKinney, M.K., Weerapana, E., Sadagopan, N., Liimatta, M., Smith, S.E., Lazerwith, S., Stiff, C., Kamtekar, S., Bhattacharya, K., Zhang, Y., Swaney, S., Van Becelaere, K., Stevens, R.C., Cravatt, B.F., 2009. Discovery and Characterization of a Highly Selective FAAH Inhibitor that Reduces Inflammatory Pain. Chem. Biol. 16, 411–420. doi:10.1016/J.CHEMBIOL.2009.02.013

Alapafuja, S.O., Nikas, S.P., Bharathan, I.T., Shukla, V.G., Nasr, M.L., Bowman, A.L., Zvonok, N., Li, J., Shi, X., Engen, J.R., Makriyannis, A., 2012. Sulfonyl Fluoride Inhibitors of Fatty Acid Amide Hydrolase. doi:10.1021/jm301205j

Alexander, J.P., Cravatt, B.F., 2006. The Putative Endocannabinoid Transport Blocker LY2183240 Is a Potent Inhibitor of FAAH and Several Other Brain Serine Hydrolases. J. Am. Chem. Soc. 128, 9699–9704. doi:10.1021/ja062999h

Bachovchin, D.A., Ji, T., Li, W., Simon, G.M., Blankman, J.L., Adibekian, A., Hoover, H., Niessen, S., Cravatt, B.F., 2010. Superfamily-wide portrait of serine hydrolase inhibition achieved by library-versus-library screening. Proc. Natl. Acad. Sci. 107, 20941–20946. doi:10.1073/pnas.1011663107

Bachovchin, D.A., Koblan, L.W., Wu, W., Liu, Y., Li, Y., Zhao, P., Woznica, I., Shu, Y., Lai, J.H., Poplawski, S.E., Kiritsy, C.P., Healey, S.E., DiMare, M., Sanford, D.G., Munford, R.S., Bachovchin, W.W., Golub, T.R., 2014. A high-throughput, multiplexed assay for superfamily-wide profiling of enzyme activity. Nat. Chem. Biol. 10, 656–663. doi:10.1038/nchembio.1578

Banijamali, A.R., Wakefield, J.D., Mermerian, A.H., Busby, R.W., 2014. Metabolism and disposition of MM-433593, a selective FAAH-1 inhibitor, in monkeys. Pharmacol. Res. Perspect. 2, e00059. doi:10.1002/prp2.59

Beltramo, M., Stella, N., Calignano, A., Lin, S.Y., Makriyannis, A., Piomelli, D., 1997. Functional role of high-affinity anandamide transport, as revealed by selective inhibition. Science 277, 1094–7.

Bisogno, T., Hanus, L., De Petrocellis, L., Tchilibon, S., Ponde, D.E., Brandi, I., Moriello, A.S., Davis, J.B., Mechoulam, R., Di Marzo, V., 2001. Molecular targets for cannabidiol and its synthetic analogues: effect on vanilloid VR1 receptors and on the cellular uptake and enzymatic hydrolysis of anandamide. Br. J. Pharmacol. 134, 845–852. doi:10.1038/sj.bjp.0704327

Chang, J.W., Niphakis, M.J., Lum, K.M., Cognetta, A.B., Wang, C., Matthews, M.L., Niessen, S., Buczynski, M.W., Parsons, L.H., Cravatt, B.F., 2012. Highly Selective Inhibitors of Monoacylglycerol Lipase Bearing a Reactive Group that Is Bioisosteric with Endocannabinoid Substrates. Chem. Biol. 19, 579–588. doi:10.1016/j.chembiol.2012.03.009

Chicca, A., Nicolussi, S., Bartholomäus, R., Blunder, M., Aparisi Rey, A., Petrucci, V., Reynoso-Moreno, I. del C., Viveros-Paredes, J.M., Dalghi Gens, M., Lutz, B., Schiöth, H.B., Soeberdt, M., Abels, C., Charles, R.-P., Altmann, K.-H., Gertsch, J., 2017. Chemical probes to potently and selectively inhibit endocannabinoid cellular reuptake. Proc. Natl. Acad. Sci. 114, E5006–E5015. doi:10.1073/pnas.1704065114

Chu, Z.-L., Carroll, C., Chen, R., Alfonso, J., Gutierrez, V., He, H., Lucman, A., Xing, C., Sebring, K., Zhou, J., Wagner, B., Unett, D., Jones, R.M., Behan, D.P., Leonard, J., 2010. N-Oleoyldopamine Enhances Glucose Homeostasis through the Activation of GPR119. Mol. Endocrinol. 24, 161–170. doi:10.1210/me.2009-0239

Cisar, J.S., Weber, O.D., Clapper, J.R., Blankman, J.L., Henry, C.L., Simon, G.M., Alexander, J.P., Jones, T.K., Ezekowitz, R.A.B., O’Neill, G.P., Grice, C.A., 2018. Identification of ABX-1431, a Selective Inhibitor of Monoacylglycerol Lipase and Clinical Candidate for Treatment of Neurological Disorders. J. Med. Chem. 61, 9062–9084. doi:10.1021/acs.jmedchem.8b00951

De Petrocellis, L., Bisogno, T., Davis, J.B., Pertwee, R.G., Di Marzo, V., 2000. Overlap between the ligand recognition properties of the anandamide transporter and the VR1 vanilloid receptor: inhibitors of anandamide uptake with negligible capsaicin-like activity. FEBS Lett. 483, 52–6.

Edgemond, W.S., Greenberg, M.J., McGinley, P.J., Muthian, S., Campbell, W.B., Hillard, C.J., 1998. Synthesis and characterization of diazomethylarachidonyl ketone: an irreversible inhibitor of N-arachidonylethanolamine amidohydrolase. J. Pharmacol. Exp. Ther. 286, 184–90.

Fegley, D., Kathuria, S., Mercier, R., Li, C., Goutopoulos, A., Makriyannis, A., Piomelli, D., 2004. Anandamide transport is independent of fatty-acid amide hydrolase activity and is blocked by the hydrolysis-resistant inhibitor AM1172. Proc. Natl. Acad. Sci. 101, 8756–8761. doi:10.1073/pnas.0400997101

Fowler, C.J., Tiger, G., Ligresti, A., López-Rodrı́guez, M.L., Di Marzo, V., 2004. Selective inhibition of anandamide cellular uptake versus enzymatic hydrolysis—a difficult issue to handle. Eur. J. Pharmacol. 492, 1–11. doi:10.1016/J.EJPHAR.2004.03.048

Genet, C., Strehle, A., Schmidt, C., Boudjelal, G., Lobstein, A., Schoonjans, K., Souchet, M., Auwerx, J., Saladin, R., Wagner, A., 2010. Structure−Activity Relationship Study of Betulinic Acid, A Novel and Selective TGR5 Agonist, and Its Synthetic Derivatives: Potential Impact in Diabetes. J. Med. Chem. 53, 178–190. doi:10.1021/jm900872z

Giang, D.K., Cravatt, B.F., Glasnapp, S., Lin, S.Y., Goutopoulos, A., Xie, X.Q., Makriyannis, A., 1997. Molecular characterization of human and mouse fatty acid amide hydrolases. Proc. Natl. Acad. Sci. 94, 2238–2242. doi:10.1073/pnas.94.6.2238

Griebel, G., Stemmelin, J., Lopez-Grancha, M., Fauchey, V., Slowinski, F., Pichat, P., Dargazanli, G., Abouabdellah, A., Cohen, C., Bergis, O.E., 2018. The selective reversible FAAH inhibitor, SSR411298, restores the development of maladaptive behaviors to acute and chronic stress in rodents. Sci. Rep. 8, 2416. doi:10.1038/s41598-018-20895-z

Ho, W.S. V, Hillard, C.J., 2005. Modulators of endocannabinoid enzymic hydrolysis and membrane transport. Handb. Exp. Pharmacol. 187–207.

Hohmann, A.G., Suplita, R.L., Bolton, N.M., Neely, M.H., Fegley, D., Mangieri, R., Krey, J.F., Michael Walker, J., Holmes, P. V., Crystal, J.D., Duranti, A., Tontini, A., Mor, M., Tarzia, G., Piomelli, D., 2005. An endocannabinoid mechanism for stress-induced analgesia. Nature 435, 1108–1112. doi:10.1038/nature03658

Hsu, K.-L., Tsuboi, K., Chang, J.W., Whitby, L.R., Speers, A.E., Pugh, H., Cravatt, B.F., 2013. Discovery and Optimization of Piperidyl-1,2,3-Triazole Ureas as Potent, Selective, and in Vivo-Active Inhibitors of α/β-Hydrolase Domain Containing 6 (ABHD6). J. Med. Chem. 56, 8270–8279. doi:10.1021/jm400899c

Huang, S.M., Bisogno, T., Petros, T.J., Chang, S.Y., Zavitsanos, P.A., Zipkin, R.E., Sivakumar, R., Coop, A., Maeda, D.Y., De Petrocellis, L., Burstein, S., Di Marzo, V., Walker, J.M., 2001. Identification of a new class of molecules, the arachidonyl amino acids, and characterization of one member that inhibits pain. J. Biol. Chem. 276, 42639–44. doi:10.1074/jbc.M107351200

Janssen, F.J., Deng, H., Baggelaar, M.P., Allarà, M., van der Wel, T., den Dulk, H., Ligresti, A., van Esbroeck, A.C.M., McGuire, R., Di Marzo, V., Overkleeft, H.S., van der Stelt, M., 2014. Discovery of Glycine Sulfonamides as Dual Inhibitors of *sn* -1-Diacylglycerol Lipase α and α/β-Hydrolase Domain 6. J. Med. Chem. 57, 6610–6622. doi:10.1021/jm500681z

Jonsson, K.-O., Vandevoorde, S., Lambert, D.M., Tiger, G., Fowler, C.J., 2001. Effects of homologues and analogues of palmitoylethanolamide upon the inactivation of the endocannabinoid anandamide. Br. J. Pharmacol. 133, 1263–1275. doi:10.1038/sj.bjp.0704199

Kaczocha, M., Rebecchi, M.J., Ralph, B.P., Teng, Y.-H.G., Berger, W.T., Kaczocha, M., Rebecchi, M.J., Ralph, B.P., Berger, Y.G., 2014. Inhibition of Fatty Acid Binding Proteins Elevates Brain Anandamide Levels and Produces Analgesia. PLoS One 9, 94200. doi:10.1371/journal.pone.0094200

Karbarz, M.J., Luo, L., Chang, L., Tham, C.-S., Palmer, J.A., Wilson, S.J., Wennerholm, M.L., Brown, S.M., Scott, B.P., Apodaca, R.L., Keith, J.M., Wu, J., Breitenbucher, J.G., Chaplan, S.R., Webb, M., 2009. Biochemical and Biological Properties of 4-(3-phenyl-[1,2,4] thiadiazol-5-yl)-piperazine-1-carboxylic acid phenylamide, a Mechanism-Based Inhibitor of Fatty Acid Amide Hydrolase. Anesth. Analg. 108, 316–329. doi:10.1213/ane.0b013e31818c7cbd

Kathuria, S., Gaetani, S., Fegley, D., Valiño, F., Duranti, A., Tontini, A., Mor, M., Tarzia, G., Rana, G. La, Calignano, A., Giustino, A., Tattoli, M., Palmery, M., Cuomo, V., Piomelli, D., 2003. Modulation of anxiety through blockade of anandamide hydrolysis. Nat. Med. 9, 76–81. doi:10.1038/nm803

Keith, J.M., Apodaca, R., Tichenor, M., Xiao, W., Jones, W., Pierce, J., Seierstad, M., Palmer, J., Webb, M., Karbarz, M., Scott, B., Wilson, S., Luo, L., Wennerholm, M., Chang, L., Brown, S., Rizzolio, M., Rynberg, R., Chaplan, S., Breitenbucher, J.G., 2012. Aryl Piperazinyl Ureas as Inhibitors of Fatty Acid Amide Hydrolase (FAAH) in Rat, Dog, and Primate. ACS Med. Chem. Lett. 3, 823–827. doi:10.1021/ml300186g

Keith, J.M., Apodaca, R., Xiao, W., Seierstad, M., Pattabiraman, K., Wu, J., Webb, M., Karbarz, M.J., Brown, S., Wilson, S., Scott, B., Tham, C.-S., Luo, L., Palmer, J., Wennerholm, M., Chaplan, S., Breitenbucher, J.G., 2008. Thiadiazolopiperazinyl ureas as inhibitors of fatty acid amide hydrolase. Bioorg. Med. Chem. Lett. 18, 4838–4843. doi:10.1016/j.bmcl.2008.07.081

Keith, J.M., Jones, W.M., Tichenor, M., Liu, J., Seierstad, M., Palmer, J.A., Webb, M., Karbarz, M., Scott, B.P., Wilson, S.J., Luo, L., Wennerholm, M.L., Chang, L., Rizzolio, M., Rynberg, R., Chaplan, S.R., Breitenbucher, J.G., 2015. Preclinical Characterization of the FAAH Inhibitor JNJ-42165279. ACS Med. Chem. Lett. 6, 1204–1208. doi:10.1021/acsmedchemlett.5b00353

Kodani, S.D., Wan, D., Wagner, K.M., Hwang, S.H., Morisseau, C., Hammock, B.D., 2018. Design and Potency of Dual Soluble Epoxide Hydrolase/Fatty Acid Amide Hydrolase Inhibitors. ACS Omega 3, 14076–14086. doi:10.1021/acsomega.8b01625

Lee, S.A., Hwang, J.S., Han, X.H., Lee, C., Lee, M.H., Choe, S.G., Hong, S.S., Lee, D., Lee, M.K., Hwang, B.Y., 2008. Methylpiperate derivatives from Piper longum and their inhibition of monoamine oxidase. Arch. Pharm. Res. 31, 679–683. doi:10.1007/s12272-001-1212-7

Li, W., Blankman, J.L., Cravatt, B.F., 2007. A Functional Proteomic Strategy to Discover Inhibitors for Uncharacterized Hydrolases. J. Am. Chem. Soc. 129, 9594–9595. doi:10.1021/ja073650c

Lichtman, A.H., Leung, D., Shelton, C.C., Saghatelian, A., Hardouin, C., Boger, D.L., Cravatt, B.F., 2004. Reversible Inhibitors of Fatty Acid Amide Hydrolase That Promote Analgesia: Evidence for an Unprecedented Combination of Potency and Selectivity. J. Pharmacol. Exp. Ther. 311, 441–448. doi:10.1124/jpet.104.069401

Ligresti, A., Cascio, M.G., Pryce, G., Kulasegram, S., Beletskaya, I., De Petrocellis, L., Saha, B., Mahadevan, A., Visintin, C., Wiley, J.L., Baker, D., Martin, B.R., Razdan, R.K., Di Marzo, V., 2006. New potent and selective inhibitors of anandamide reuptake with antispastic activity in a mouse model of multiple sclerosis. Br. J. Pharmacol. 147, 83–91. doi:10.1038/sj.bjp.0706418

Long, J.Z., Li, W., Booker, L., Burston, J.J., Kinsey, S.G., Schlosburg, J.E., Pavón, F.J., Serrano, A.M., Selley, D.E., Parsons, L.H., Lichtman, A.H., Cravatt, B.F., 2009. Selective blockade of 2-arachidonoylglycerol hydrolysis produces cannabinoid behavioral effects. Nat. Chem. Biol. 5, 37–44. doi:10.1038/nchembio.129

Long, J.Z., Nomura, D.K., Vann, R.E., Walentiny, D.M., Booker, L., Jin, X., Burston, J.J., Sim-Selley, L.J., Lichtman, A.H., Wiley, J.L., Cravatt, B.F., 2009. Dual blockade of FAAH and MAGL identifies behavioral processes regulated by endocannabinoid crosstalk in vivo. Proc. Natl. Acad. Sci. 106, 20270–20275. doi:10.1073/pnas.0909411106

López-Rodríguez, M.L., Viso, A., Ortega-Gutiérrez, S., Fowler, C.J., Tiger, G., de Lago, E., Fernández-Ruiz, J., Ramos, J.A., 2003. Design, synthesis and biological evaluation of new endocannabinoid transporter inhibitors. Eur. J. Med. Chem. 38, 403–12.

Marrs, W.R., Horne, E.A., Ortega-Gutierrez, S., Cisneros, J.A., Xu, C., Lin, Y.H., Muccioli, G.G., Lopez-Rodriguez, M.L., Stella, N., 2011. Dual Inhibition of α/β-Hydrolase Domain 6 and Fatty Acid Amide Hydrolase Increases Endocannabinoid Levels in Neurons. J. Biol. Chem. 286, 28723–28728. doi:10.1074/jbc.M110.202853

Martin, B.R., Beletskaya, I., Patrick, G., Jefferson, R., Winckler, R., Deutsch, D.G., Di Marzo, V., Dasse, O., Mahadevan, A., Razdan, R.K., 2000. Cannabinoid properties of methylfluorophosphonate analogs. J. Pharmacol. Exp. Ther. 294, 1209–18.

McHugh, D., Hu, S.S.J., Rimmerman, N., Juknat, A., Vogel, Z., Walker, J.M., Bradshaw, H.B., 2010. N-arachidonoyl glycine, an abundant endogenous lipid, potently drives directed cellular migration through GPR18, the putative abnormal cannabidiol receptor. BMC Neurosci. 11, 44. doi:10.1186/1471-2202-11-44

Migliore, M., Habrant, D., Sasso, O., Albani, C., Bertozzi, S.M., Armirotti, A., Piomelli, D., Scarpelli, R., 2016. Potent multitarget FAAH-COX inhibitors: Design and structure-activity relationship studies. Eur. J. Med. Chem. 109, 216–237. doi:10.1016/j.ejmech.2015.12.036

Moore, S.A., Nomikos, G.G., Dickason-Chesterfield, A.K., Schober, D.A., Schaus, J.M., Ying, B.-P., Xu, Y.-C., Phebus, L., Simmons, R.M.A., Li, D., Iyengar, S., Felder, C.C., 2005. Identification of a high-affinity binding site involved in the transport of endocannabinoids. Proc. Natl. Acad. Sci. U. S. A. 102, 17852–7. doi:10.1073/pnas.0507470102

Navia-Paldanius, D., Savinainen, J.R., Laitinen, J.T., 2012. Biochemical and pharmacological characterization of human α/β-hydrolase domain containing 6 (ABHD6) and 12 (ABHD12). J. Lipid Res. 53, 2413–2424. doi:10.1194/jlr.M030411

Nicolussi, S., Viveros-Paredes, J.M., Gachet, M.S., Rau, M., Flores-Soto, M.E., Blunder, M., Gertsch, J., 2014. Guineensine is a novel inhibitor of endocannabinoid uptake showing cannabimimetic behavioral effects in BALB/c mice. Pharmacol. Res. 80, 52–65. doi:10.1016/j.phrs.2013.12.010

Niforatos, W., Zhang, X.-F., Lake, M.R., Walter, K.A., Neelands, T., Holzman, T.F., Scott, V.E., Faltynek, C.R., Moreland, R.B., Chen, J., 2007. Activation of TRPA1 Channels by the Fatty Acid Amide Hydrolase Inhibitor 3’-Carbamoylbiphenyl-3-yl cyclohexylcarbamate (URB597). Mol. Pharmacol. 71, 1209–1216. doi:10.1124/mol.106.033621

Niphakis, M.J., Cognetta, A.B., Chang, J.W., Buczynski, M.W., Parsons, L.H., Byrne, F., Burston, J.J., Chapman, V., Cravatt, B.F., 2013. Evaluation of NHS Carbamates as a Potent and Selective Class of Endocannabinoid Hydrolase Inhibitors. ACS Chem. Neurosci. 4, 1322–1332. doi:10.1021/cn400116z

Ortar, G., Ligresti, A., De Petrocellis, L., Morera, E., Di Marzo, V., 2003. Novel selective and metabolically stable inhibitors of anandamide cellular uptake. Biochem. Pharmacol. 65, 1473–1481. doi:10.1016/S0006-2952(03)00109-6

Parkkari, T., Haavikko, R., Laitinen, T., Navia-Paldanius, D., Rytilahti, R., Vaara, M., Lehtonen, M., Alakurtti, S., Yli-Kauhaluoma, J., Nevalainen, T., Savinainen, J.R., Laitinen, J.T., 2014. Discovery of Triterpenoids as Reversible Inhibitors of α/β-hydrolase Domain Containing 12 (ABHD12). PLoS One 9, e98286. doi:10.1371/journal.pone.0098286

Patel, J.Z., Nevalainen, T.J., Savinainen, J.R., Adams, Y., Laitinen, T., Runyon, R.S., Vaara, M., Ahenkorah, S., Kaczor, A.A., Navia-Paldanius, D., Gynther, M., Aaltonen, N., Joharapurkar, A.A., Jain, M.R., Haka, A.S., Maxfield, F.R., Laitinen, J.T., Parkkari, T., 2015. Optimization of 1,2,5-thiadiazole carbamates as potent and selective ABHD6 inhibitors. ChemMedChem 10, 253–65. doi:10.1002/cmdc.201402453

Patel, J.Z., van Bruchem, J., Laitinen, T., Kaczor, A.A., Navia-Paldanius, D., Parkkari, T., Savinainen, J.R., Laitinen, J.T., Nevalainen, T.J., 2015. Revisiting 1,3,4-Oxadiazol-2-ones: Utilization in the Development of ABHD6 Inhibitors. Bioorg. Med. Chem. 23, 6335–6345. doi:10.1016/j.bmc.2015.08.030

Pawsey, S., Wood, M., Browne, H., Donaldson, K., Christie, M., Warrington, S., 2016. Safety, Tolerability and Pharmacokinetics of FAAH Inhibitor V158866: A Double-Blind, Randomised, Placebo-Controlled Phase I Study in Healthy Volunteers. Drugs R. D. 16, 181–191. doi:10.1007/s40268-016-0127-y

Piomelli, D., Beltramo, M., Glasnapp, S., Lin, S.Y., Goutopoulos, A., Xie, X.Q., Makriyannis, A., 1999. Structural determinants for recognition and translocation by the anandamide transporter. Proc. Natl. Acad. Sci. U. S. A. 96, 5802–7.

Riendeau, D., Guay, J., Weech, P.K., Laliberté, F., Yergey, J., Li, C., Desmarais, S., Perrier, H., Liu, S., Nicoll-Griffith, D., 1994. Arachidonyl trifluoromethyl ketone, a potent inhibitor of 85-kDa phospholipase A2, blocks production of arachidonate and 12-hydroxyeicosatetraenoic acid by calcium ionophore-challenged platelets. J. Biol. Chem. 269, 15619–24.

Saario, S.M., Salo, O.M.H., Nevalainen, T., Poso, A., Laitinen, J.T., Järvinen, T., Niemi, R., 2005. Characterization of the Sulfhydryl-Sensitive Site in the Enzyme Responsible for Hydrolysis of 2- Arachidonoyl-Glycerol in Rat Cerebellar Membranes. Chem. Biol. 12, 649–656. doi:10.1016/j.chembiol.2005.04.013

Tuo, W., Leleu-Chavain, N., Spencer, J., Sansook, S., Régis, R., Chavatte, P., 2016. Therapeutic Potential of Fatty Acid Amide Hydrolase, Monoacylglycerol Lipase, and N-Acylethanolamine Acid Amidase Inhibitors. doi:10.1021/acs.jmedchem.6b00538

Tzviel Sheskin, †, Lumir Hanuš, †, Joram Slager, †, Zvi Vogel, ‡ and, Raphael Mechoulam*, †, 1997. Structural Requirements for Binding of Anandamide-Type Compounds to the Brain Cannabinoid Receptor. doi:10.1021/JM960752X

van Esbroeck, A.C.M., Janssen, A.P.A., Cognetta, A.B., Ogasawara, D., Shpak, G., van der Kroeg, M., Kantae, V., Baggelaar, M.P., de Vrij, F.M.S., Deng, H., Allarà, M., Fezza, F., Lin, Z., van der Wel, T., Soethoudt, M., Mock, E.D., den Dulk, H., Baak, I.L., Florea, B.I., Hendriks, G., De Petrocellis, L., Overkleeft, H.S., Hankemeier, T., De Zeeuw, C.I., Di Marzo, V., Maccarrone, M., Cravatt, B.F., Kushner, S.A., van der Stelt, M., 2017. Activity-based protein profiling reveals off-target proteins of the FAAH inhibitor BIA 10-2474. Science (80-. ). 356, 1084–1087. doi:10.1126/science.aaf7497

Watabiki, T., Tsuji, N., Kiso, T., Ozawa, T., Narazaki, F., Kakimoto, S., 2017. In vitro and in vivo pharmacological characterization of ASP8477: A novel highly selective fatty acid amide hydrolase inhibitor. Eur. J. Pharmacol. 815, 42–48. doi:10.1016/J.EJPHAR.2017.10.007
